# Emerging nanozyme-based multimodal synergistic therapies in combating bacterial infections

**DOI:** 10.7150/thno.73681

**Published:** 2022-08-08

**Authors:** Yanmei Zhang, Xin Hu, Jing Shang, Wenhui Shao, Liming Jin, Chunshan Quan, Jun Li

**Affiliations:** 1 College of Life Science, Dalian Minzu University, Economical and Technological Development Zone, Dalian, 116600, China.; 2 State Key Laboratory of Catalysis, Dalian Institute of Chemical Physics, Chinese Academy of Science, P. O. Box 110, Dalian 116023, China.; 3 Key Laboratory of Biotechnology and Bioresources Utilization (Dalian Minzu University), Ministry of Education, China.

**Keywords:** Multidrug resistance, nanozyme, peroxidase, oxidase, reactive oxygen radical, photothermal effect, selective, integrated platform, detection

## Abstract

Pathogenic infections have emerged as major threats to global public health. Multidrug resistance induced by the abuse of antibiotics makes the anti-infection therapies to be a global challenge. Thus, it is urgent to develop novel, efficient and biosafe antibiotic alternatives for future antibacterial therapy. Recently, nanozymes have emerged as promising antibiotic alternatives for combating bacterial infections. More significantly, the multimodal synergistic nanozyme-based antibacterial systems open novel disinfection pathways. In this review, we are mainly focusing on the recent research progress of nanozyme-based multimodal synergistic therapies to eliminate bacterial infections. Their antibacterial mechanism, the synergistic antibacterial systems are systematically summarized and discussed according to the combination of mechanisms and the purpose to improve their antibacterial efficiency, biosafety and specificity. Finanly, the current challenges and prospects of the multimodal synergistic antibacterial systems are proposed.

## 1. Introduction

In recent decade, bacterial infections are being recognized as a serious public threat to public health. Many conventional natural or chemically synthesized antibiotics can selectively inhibit or kill bacterium. However, pathogens can adapt or become resistant to almost all available traditional antibiotics and evolved into multi drug-resistant strains even super bacteria, threatening the survivals of millions of people in the global word. The emergence of “super bacteria” not only greatly reduced the clinical effect of bacterial infection treatment, but also caused the dilemma of “no medicine available” 1,2. In addition, the clinical treatment of biofilm-related infections is more difficult because biofilm is a three-dimensional structure formed by microorganisms attached to biological or non-biological surfaces in which cells are surrounded by extracellular polymers produced by themselves. Protective-biofilm formation is considered as a self-defense mechanism produced by microorganisms in response to biological and non-biological stresses. Thus, it is difficult for common drugs to kill bacteria encapsulated in biofilm by extracellular matrix, which leads to higher resistance of bacteria in biofilm form. Compared with planktonic-bacteria without biofilm, biofilm-bacteria are much more recalcitrant to antibiotics, hydrogen peroxide (H_2_O_2_) and other antimicrobial agents 3. Therefore, it is highly desirable to develop new antimicrobial agents or strategies with high efficiency, environmental friendliness and without inducing drug-resistance for combating planktonic-bacteria and biofilms.

**Figure 1:**
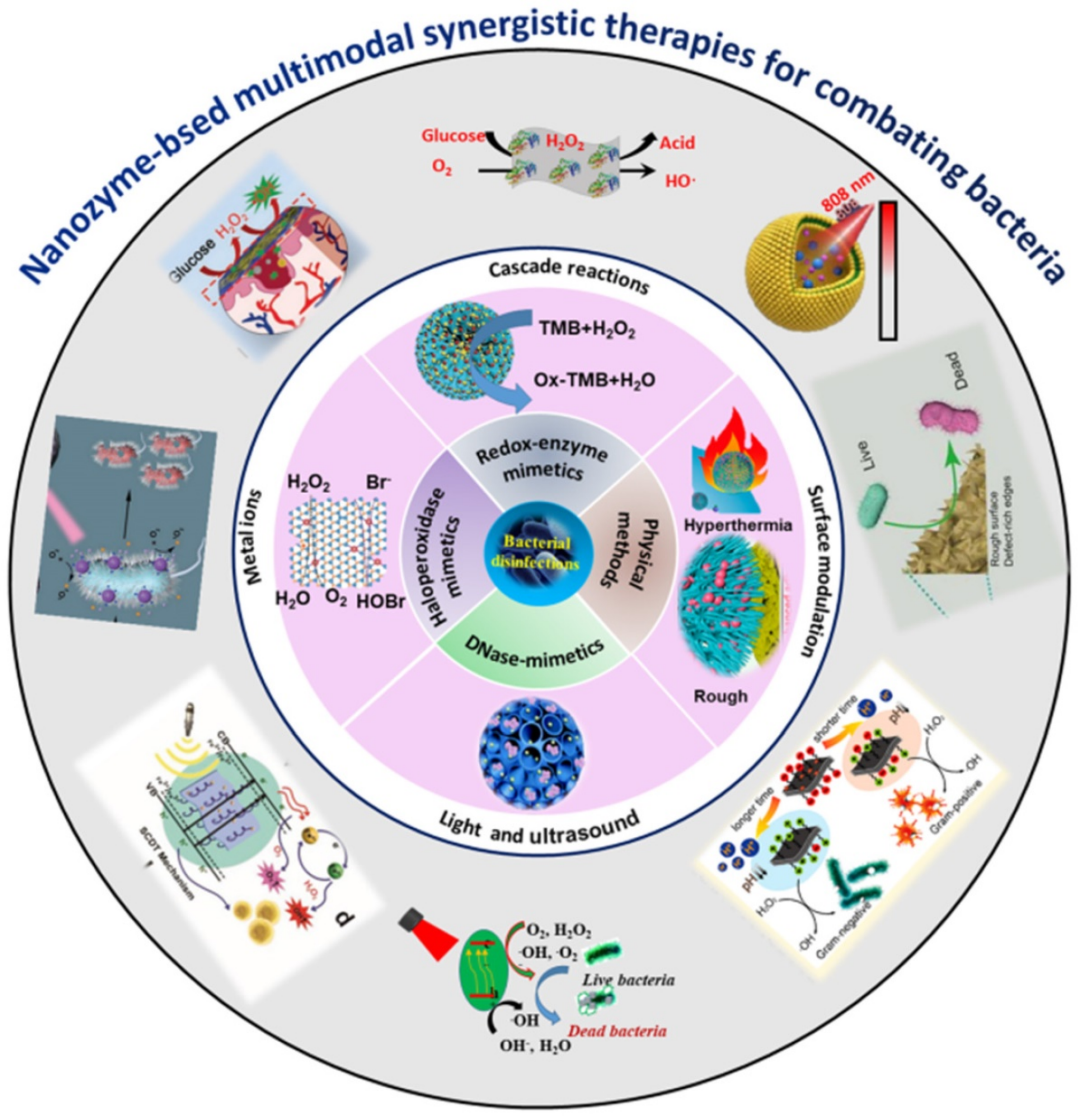
Schematic diagram of nanozyme-based synergistic antibacterial strategies for improved antimicrobial performances and biosafety. Partial images were adapted with permission from ref. 29, copyright 2022, ref. 32, copyright 2019, ref. 33, copyright 2019, ref. 34, copyright 2020, ref. 38, copyright 2021, ref. 39, copyright 2019, WILEY-VCH. Ref. 30, copyright 2021, ref. 31, copyright 2019, ref. 36, copyright 2020, ref. 37, copyright 2018, the American Chemical Society. Ref. 35, copyright 2020, ref. 40, copyright 2019, the Royal Society of Chemistry.

To eliminate the increasing threat of drug-resistance bacterial infections, extensive interdisciplinary efforts have been devoted to discovery and design of novel and more efficient antimicrobial agents. The rapid development of nanozymes provides us new opportunities to solve the problems 4-10. Nanozymes are refer to artificial nanomaterials with intrinsic enzyme-like catalytic performance and have arouse wide concerns in the recent decades. Unlike natural enzymes, nanozymes are low cost, more stable and activity-tunable, which are considered to be promising alternatives to natural enzymes for applications in industrial, biological, and medical fields 4-19. Compared with traditional antibiotics, nanozymes have higher membrane permeability and can be used as an inhibitor of efflux pump. Moreover, it is not easy to induce drug resistance because they can achieve multiple antibacterial functions. Importantly, the antibacterial performance is tunable by controlling the size, structure, surface morphology, components, surface charge and other factors of nanomaterials, and can be further optimized by combining more than one functional mechanism in one 4-40. In particular, in addition to their own antibacterial ability, many nanozyme-based antibacterial agents have the characteristics of magnetism, photothermal, fluorescence 41-44. Therefore, nanozymes-based multimodal synergistic platforms for combating bacterial infections can be constructed by combining them with their antibacterial activities, and thus realize the separation, detection and killing of bacteria in one 21,24-26. To date, much attractive progress in this regard has been reported, thus comprehensive reviews are highly desirable towards their current progress. Herein, in this comprehensive review, we have mainly focused on summary of the recent advances of multimodal synergistic antibacterial strategies (Figure 1). Finally, the current challenges and prospect of nanozyme-based synergistic antibacterial strategies in practical antibacterial applications are discussed. This review is intended to provide new insights into developing new safe antibacterial therapeutics for clinical applications.

## 2. Antibacterial Mechanisms of nanozymes

In recent decade, nanozyme-based antibacterial agents have become a hotspot in antibacterial field. Compared with traditional antibiotics, they can execute multiple bactericidal pathways thus is not easy to induce drug-resistance for combating bacteria and biofilms 45-50. Up to now, a significant number of nanomaterials such as carbons 51,52, noble metal nanoparticles 53-55, metal organic frameworks (MOFs) 31, 33,46, covalent organic frameworks (COFs) 38,56,57 and metal oxides 58-61 have been discovered and designed that exhibit peroxidase (POD) 62-65, oxidase (OXD) 55,65-68, deoxyribonuclease (DNase) 69,70 and haloperoxidase (HPO) 71-74 mimetic activities. Currently, by utilizing their superior performances, the nanozyme-based antibacterial agents have emerged as new powerful tools for combating bacterial infections. Their antibacterial mechanisms are summarized as follows. In particular, in this review, we also termed the nanomaterials that can activate molecular oxygen especially under light or ultrasound into reactive oxygen species as redox nanozymes, even though the authors didn't refer to their biomimetic properties in their articles.

### 2.1 Combating bacterial infections by redox enzyme mimetics

Due to the super oxidation capability of high level of reactive oxygen species (ROS) which can damage the lipids, proteins and DNA in the biofilm matrix, on the cell surface and inside the microbial cells, both planktonic cells and biofilms can be killed and eliminated when exposed to high level of ROS 75-81. Therefore, it is not easy to induce drug resistance. As known, H_2_O_2_ alone at a higher concentration possess good antibacterial performance and serve as a general antibacterial agent. However, it is not safe for healthy tissues in the presence of high concentration of H_2_O_2_. Fortunately, POD-like nanozymes can produced highly oxidative •OH by decomposing very low concentration of H_2_O_2_ to achieve higher antibacterial efficacy without toxicity to healthy tissues 63-65,81,82. By virtue of this ability, ferromagnetic nanoparticles were used as a POD-like for oxidative biofilm elimination in the presence of H_2_O_2_ 63. Later, graphene quantum dots (GQDs) were also put forward as safe POD-like mimetics for actual wound disinfection with H_2_O_2_ at low dose 81. Apart from the aforementioned POD-mimetic, some nanomaterials exhibit OXD-mimetic activity that can directly activate oxygen to generate ROS like H_2_O_2_, superoxide anions and single oxygen spontaneously which can kill bacteria and eradicate their biofilms 83-91. By virtue of this ability, many OXD nano-mimetics such as Tb4O7 nanoparticles 88, silica-supported AuNPs 65, Pd nanoparticles 53, Pt/Ag nanoalloys 89 exhibit bactericidal activities in the absence of H_2_O_2_. Furthermore, many nanomaterials exhibit multi enzyme-like activities 29,53,65,89-91. For example, Cu_2_WS_4_ nanocrystals exhibit excellent OXD-like and POD-like activities to generate ROS and act as efficient antibacterial agents for healing mouse skin infections 91.

### 2.2 Combating bacterial infections by DNase-mimetic artificial enzyme

Extracellular DNA (eDNA) is one of important extracellular polymeric substance (EPS) constituents for many bacterial species and has a very significant role in maintaining the biofilm integrity. DNase mimetics can cleave eDNA to inhibit the formation of biofilms and disperse the established biofilms 69,70. Recently, Qu et al. designed and synthesized bifunctional nanozyme-based synergistic systems by integrating DNase-like and POD-like mimetics for combating biofilms 69. The bifunctional MOF/Ce-based nanozymes are capable of hydrolyzing eDNA and disrupting established biofilms by the cerium (IV) complexes (DNase mimetics), and kill bacteria exposed in dispersed biofilms by POD-like MOF in the presence of H_2_O_2_ 69.

### 2.3 Combating bacterial infections by HPO-like mimetics

HPO mimetics can prevent biofilm growth and disrupt biofilms by blocking quorum sensing through quenching auto-inducers (signal molecules related to quorum sensing) 71-74. For example, vanadium pentoxide (V_2_O_5_) nanomaterials showing HPO-mimetic properties have been shown to function as biocatalytic bactericidal nano-agents with the assistance of bromide ions (Br^-^) and H_2_O_2_ via the bromination process 73. As a result, compared to the absence of additives, V_2_O_5_ nanowires could inhibit the bacterial growth more efficiently with the assistance of Br^-^ and H_2_O_2_. Significant decreases in bacterial growth were observed (the decrease is 78% for *E. coli* and 96% for *S. aureus*). Typically, this approach is efficient for bacterial disinfection in mild alkaline environments rather than in acidic environments, thus can be used to marine antifouling. In another work, it was found that CeO_2_-x nanorods could inhibit the bacterial growth after only 5s of coincubation because of their morphology-dependent HPO-mimetic activity. More importantly, cerium-based nanocatalysts are more biocompatible than the vanadium-based ones, thus have more potentials in clinical antibacterial treatment 74. In order to overcome the instability of natural enzymes and supply adequate H_2_O_2_, Luo et al., presented a semiconductor-based bifunctional nanozyme for antibiofouling that can sustainably self-supply H_2_O_2_ from water and O_2_ by photosynthesis for HPO-mimicking reaction in a sequential manner 29.

### 2.4 Combating bacterial infections by near infrared (NIR) light laser-induced hyperthermia

Antibacterial photothermal therapy (PTT) has attracted great attentions for bacterial infection treatment as a non-invasive therapeutic technique because of its high efficiency, broad-spectrum antibacterial activities and negligible bacterial resistance 92-97. Generally, the photothermal agents with high light-to-heat conversion ability can adsorb NIR energy and induce localized hyperthermia, which is able to rupture membranes, damage protein and other inherent bioactive matrix, thus destruct bacteria irreversibly.

## 3. Nanozyme-based multimodal synergistic therapies in combating bacterial infections

Up to now, a variety of nanozyme-based catalytic platforms have been developed for bacterial disinfection and their biofilm eradiation 13, 18, 47. Although most of them exhibited acceptable disinfection outcomes, the clinical applications of single modal therapies are still limited because of their low therapeutic efficacy, intolerable cellular toxicity, arrow-spectrum antibacterial activities, non-targeting and short-term physiological stability. Recently, nanozyme-based synergetic systems have been commonly used for increasing the antibacterial efficiency, biosafety and selectivity. Furthermore, in comparison to single bactericidal modality, multimodal synergetic systems minimize the drug-resistance and could achieve site-specific and controlled therapies 47.

In this section, we summarized the multimodal synergistic therapies classified by mechanism and purpose: 3.1 Tuning the surface adhesive property of nanozymes to enhanced bacterial attachment; 3.2 Reducing the usage dose of H_2_O_2_ or directly activating O_2_ to produce ROS; 3.3 Combination of more than one antibacterial mechanism. 3.4 Selective or targeted disinfections; 3.5 Nanozymes for suppressing intracellular bacteria; 3.6 Multifunctional platforms for detection and inhibition of bacteria.

### 3.1 Tuning the surface adhesive property of nanozymes to enhanced bacterial attachment

By increasing the catalytic activities of nanozymes, more ROS species with high toxicity are produced, thus improving the antibacterial activity. However, ROS species are instable and their diffusion distance is very limited in the practical environment, thus most generated ROS species do not play effective roles in combating bacteria, resulting in low antibacterial efficiency and biosafety 98-104. Therefore, both enhancement of ROS-generation ability and binding capacity to bacteria are crucial to the realization of efficient antibacterial activity of nanozyme-based antibacterial agents.

**Figure 2:**
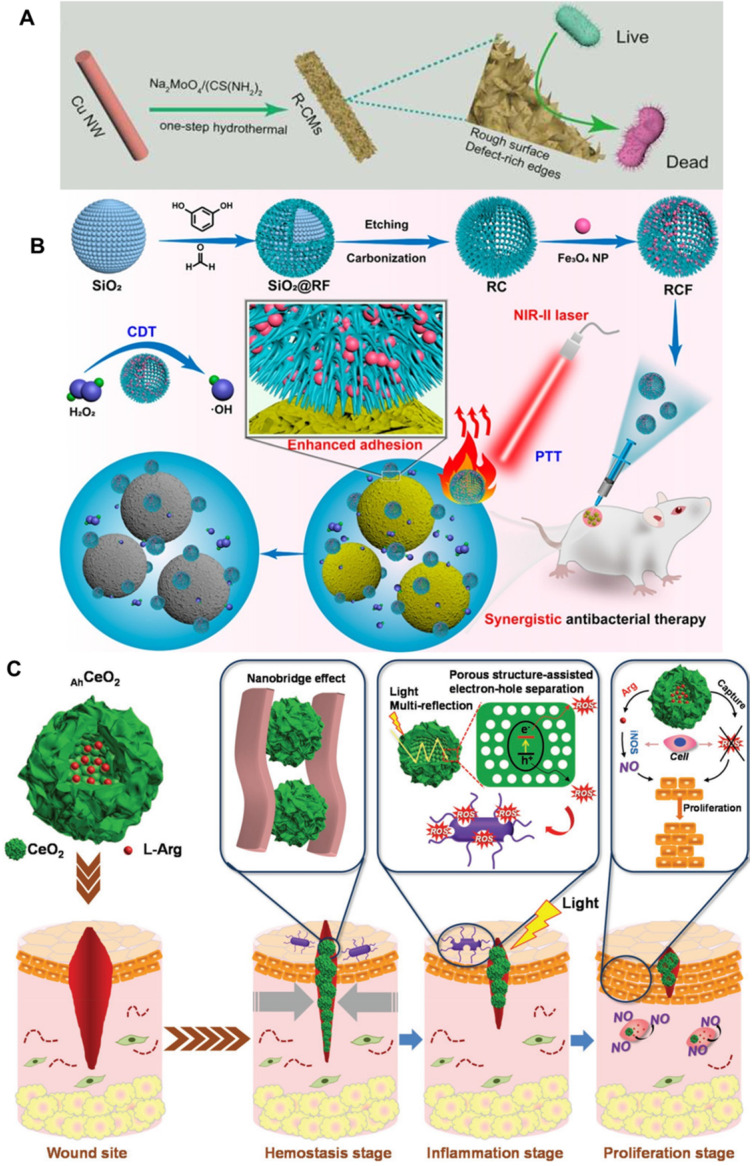
** A)** Defect-rich adhesive nanozymes with rough surface and defect-improved catalytic activity exhibited increased antibacterial activity, adapted permission from ref. 39, copyright 2019 the WILEY-VCH. **B)** Carbon-iron oxide nanohybrids with rough surface for NIR light-responsive synergistic antibacterial therapy, adapted permission from ref. 30, copyright 2021 the American Chemical Society. **C)** Hollow cerium oxide nanoparticles with rough surface promote multiple stages of wound healing, adapted permission from ref. 105, copyright 2019 the WILEY-VCH.

In this regard, Qu et al. constructed a Cu nanowires supported-MoS_2_ nanozyme with defect-rich active edges and rough surface for enhanced bacterial inhibition (Figure 2A) 39. The rough surface of the MoS_2_ endow the nanozyme with enhanced bacteria-capturing ability compared to non-adhesive MoS_2_. Furthermore, experimental and theoretical studies show that the nanozyme with defect-rich active edges has lower H_2_O_2_ adsorption energy, OH* (OH* is the adsorbed intermediates of the H_2_O_2_-decomposition process) desorption energy and greater exothermic reaction energy, which endows it with enhanced POD-like activity. In summary, the designed nanozyme has “roughness-enhanced adhesion” and “defect-improved catalytic activity”, and thus has good antibacterial effect on both two typical types of model bacteria *in vitro* and *in vivo*. Given the same nature-inspired design philosophy, Shan et al., also reported cuboid-like Cu_2_WS_4_ nanocrystals with small size for killing two model bacteria at low concentration in the absence or presence of ambient light 91. The Cu_2_WS_4_ nanocrystals exhibit OXD-like and POD-like catalytic activity thus can produce toxic ROS to kill bacteria. Impressively, the nanoparticles can selectively adsorb on the surface of bacteria because of the strong interaction between the Cu atoms on the nanozyme and amino groups from peptidoglycan in the bacteria cell wall. Thus, the cuboid-like Cu_2_WS_4_ nanocrystal with both high enzyme-like activity and selective bacterial adhesive ability endow the nanozymes with excellent antibacterial performance at very low concentration. The simultaneously generated ROS *in situ* by enzyme-like Cu_2_WS_4_ attached on the bacteria surface can efficiently kill bacteria. Furthermore, the as-prepared nanozymes have good biocompatibility and negligible toxicity, further demonstrating their promising potentials in combatting bacterial infections. For the same purpose, Yan et al. proved that Au@AgAu alloy nanoflowers with rough surface is efficient to inhibit bacteria growth with high biocompatibility 104. Very recently, Liu et al. also proved the importance of the rough surface in a carbon-iron oxide nanoparticles (Figure 2B) related synergistic antibacterial nanoplatform for effective antibacterial therapy 30. The carbon-iron oxide nanoparticles exhibit excellent POD-like activity and convert NIR light with low power density to hyperthermia efficiently, thus realize synergistic antibacterial system by combining photothermal therapy (PTT) and chemodynamic therapy (CDT). Typically, the carbon-iron oxide nanoparticles show increased bacterial adhesion due to its rough surfaces and benefit both CDT and PTT performance for broad-spectrum synergistic antibacterial effect on Gram-negative *Escherichia coli* (*E. coli*), Gram-positive* Staphylococcus aureus* (*S. aureus*), and methicillin-resistant *Staphylococcus aureus* (*MRSA*). Furthermore, the carbon-iron oxide nanoparticles show satisfactory biocompatibility and can be used as a synergistic antibacterial platform *in vivo* for healing the rat wound model infected with *MRSA*.

Hierarchical stimulation is a promising method for the fast wound closure by accelerating the complex and sequential biological process involved in the wound healing process. Ma et al. developed a compact and programmable nano-system for hierarchical stimulation to sequentially promote the hemostasis, inflammation, and proliferation stages (Figure 2C) 105. The hybrid was prepared by encapsulating L-arginine inside hollow ceria nanoparticles with rough surface. Specially, the surface roughness of the nano-system benefits the hemostasis stage by eliminating wound infection and promoting wound healing. Finally, taking advantage of enzyme-like property of the hollow ceria nanoparticles and the released L-arginine, the nano-system promote the proliferation stage. In summary, this kind of rough surface synergetic modality is simple and efficient for enhanced nanozymes induced bacterial disinfections and would healing.

### 3.2 Reducing the usage dose of H_2_O_2_ or directly activating O_2_ to produce ROS

#### 3.2.1 Cascade reactions in bacterial infection control

POD or mimetics could convert H_2_O_2_ into ROS to kill bacteria or cancer cells. However, their applications in living systems are limited because most of them exhibit optimized activity in acidic environments with a pH range of 3-4. Moreover, the introduction of high concentration of toxic H_2_O_2_ would damage normal tissues 88, 106. Recently, cascade reactions, commonly found in living organisms, are proved to be a novel powerful bacterial infection-control strategy for killing bacteria 107-120. For example, natural substrates such as glucose and oxygen in human body can be converted directly into ROS by infection-control cascade reactions to kill tumor cells and bacteria without using external toxic H_2_O_2_ 108-115, 117-120.

**Figure 3:**
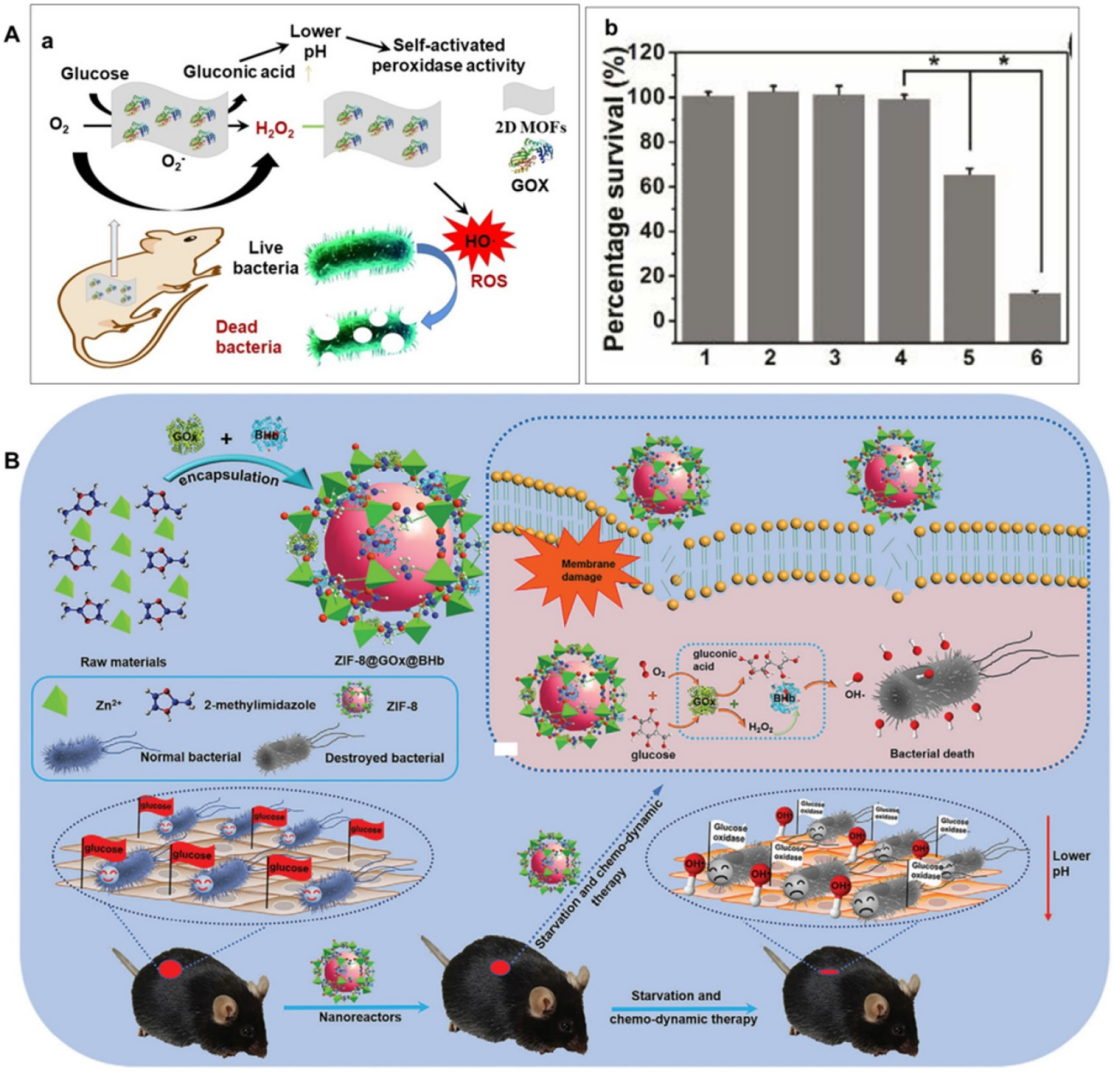
** A)** Schematic presentation of GOX/MOF hybrid nanozyme as a benign and self-activated cascade reaction platform for efficient bacteria killing and *in vivo* wound healing wound healing (a) and viability analyses of *E. coli* incubated with different systems (b). 1) PBS, 2) glucose, 3) glucose+2D MOF nanosheet, 4) 2D MOF/GOX, 5) glucose+GOX, 6) glucose+2D MOF/GOX, respectively. Adapted permission from ref. 31, copyright 2019 the American Chemical Society. **B)** Schematic preparation, mechanism and application of ZIF-8@GOX@BHb for wound infection in diabetic mice through a cascaded catalytic reaction, adapted permission from ref. 120, Copyright 2021, the WILEY-VCH.

Glucose, one of the most important carbohydrates in the human body, can be oxidized by O_2_ to produce gluconic acid and H_2_O_2_ catalyzed by GOX or mimetics through a reductive and oxidative half-reaction 108-110. Furthermore, H_2_O_2_ can be transformed into ROS by various peroxidase or mimic, such as metal organic frameworks, Fe_3_O_4_, Ir nanoplate, Au NPs, Pt and carbon nitride 28, 63-65, 106, 111. Based on these considerations, as demonstrated in Figure 3A(a), Qu et al. selected ultra-thin two-dimensional MOF nanosheet as a POD-like mimic and simultaneously physically adsorb GOX on it to manufacture a hybrid nano catalyst as a safe, self-activated cascade platform for killing bacteria and healing wound *in vivo* 31. In this system, GOX can continuously convert non-toxic glucose into gluconic acid and H_2_O_2_. Therefore, the system can act efficiently without introducing external toxic H_2_O_2_ with minimal harmful side effects. Meanwhile, the resulting gluconic acid can induce the environment from neutral to medium acidic, which can significantly enhance the POD-like activity of the two-dimensional MOF nanosheet and generate sufficient toxic hydroxyl radials to effectively eliminate bacteria. As shown in Figure 3A(b), compared the other 5 groups, the group incubated with glucose+2D MOF/GOX exhibited the highest killing efficiency against *E. coli* and *S. aureus*. Furthermore, the cascade reaction-based platform could effectively eliminate bacteria *in vitro* and *in vivo* with negligible biological toxicity 31.

To overcome limitation of the extra usage of H_2_O_2_, Tong's group reports an microreactors including both GOX and hemoglobin (Hb) inside a multilayer film as an enzymatic cascade reaction-based antibacterial system for inhibiting bacterial growth and biofilm formation 115. For the same purpose, another cascade reaction-based antibacterial system was fabricated by introducing CaO_2_ and hemin-containing graphene into alginate nanocarriers 116. During the reaction, ROS were generated by local converse of CaO_2_ and H_2_O through a localized cascade reaction. This H_2_O_2_-free depots has great potential in eradicating biofilm *in vivo*. Li et al. also developed a glucose-triggered cascade strategy for sterilization and healing wound infection. The loaded GOX in the ionic covalent-organic framework (COF) could convert glucose to H_2_O_2_, which could be decomposed directly to be more toxic hydroxyl radicals catalyzed by the pH-dependent POD-like activity of ionic covalent-organic framework-based nanozyme (GFeF) enhanced by the produced gluconic acid. Furthermore, the positively charged GFeF shows stronger adhesion ability to negatively charged bacterial membrane and thus greatly enhanced wound healing effects 38. This strategy has great potential in the wound healing of diabetic patients without harmful side effects. Similarly, Zhao's group innovatively prepared an Au-Au/IrO_2_@Cu(PABA) nanocomposite with tandem enzyme-mimicking catalytic activity simultaneously under neutral conditions and utilized in a glucose-triggered cascade strategy for efficient antibacterial application 117. Inspired by the natural multi-enzyme system, Kang group designed a well-designed L-Arg/GOX@CuBDC biomimetic composite for achieving the synergistic antibacterial effect with good biocompatibility through a double cascade reaction 118. To further increase the antibacterial efficiency, Wang et al., developed a triple-synergistic strategy for efficient bacterial eradication. In the triple-synergistic antibacterial system, ZIF-8 was used as carrier to integrate gold nanoparticles (Au NPs) and GOX into a cascade reaction to convert glucose into ROS. Typically, the acidic environment generated by the production of gluconic acid facilitate both the ROS production and leaching of Zn^2+^, thus achieved enhanced overall bacterial eradication at a low dosage for both *S. aureus* and *E. coli*. This triple-synergistic strategy exhibits great potential in bacterial eradication and biomedical applications 119.

It is imminent and challenging to develop novel antibacterial agent for killing multidrug-resistant (MDR) bacteria and healing of the diabetic wound where contain more glucose. Thus, diabetic wound healing is still a big challenge. To overcome this problem, as shown in Figure 3B, Niu's group constructed a cascaded nanoreactors (ZIF-8@GOX@BHb) by encapsulating both GOX and (bovine hemoglobin) BHb into porous ZIF-8 by a spatial confinement effect. Benefiting from the two types of enhanced enzyme-like activities and effective cascaded catalytic antibacterial performance of the obtained ZIF-8@GOX@BHb, the cascaded nanoreactors could starve and kill MDR bacteria efficiently. Impressively, the constructed nanoreactors is degradable and biocompatible for combating multidrug-resistant bacteria *in vitro* and diabetic wound healing *in vivo* 120.

Recently, to avoid the direct use of extra H_2_O_2_ and low pH medium, glucose dependent cascade reactions were used to eradicate bacteria by converting glucose into H_2_O_2_ which further decomposed into toxic ROS catalyzed by the acidic environment promoted POD-like nanozyme 118,119. However, low oxygen content in the anoxic microenvironment of the diabetic wound limit glucose-dependent cascade reaction. To address this, Li et al., designed a defect-rich MoS_2_@Au@BSA nanozyme attached injectable hydrogels with high multi-enzyme catalytic activities for diabetic wound healing through an O_2_ self-supplying cascade reaction. The nano-system can catalyze glucose into H_2_O_2_ and gluconic acid, which can be converted into ROS by the acid-enhanced POD-like activity of MoS_2_@Au@BSA for bacteria eradication. Specially, MoS_2_@Au@BSA exhibit superoxide dismutase-like activity at an alkalescent condition to transform superoxide anions into dioxygen and H_2_O_2_, and catalase-like mimetics to decomposes endogenous and exogenous H_2_O_2_ into O_2_, thus facilitate glucose oxidation and accelerates diabetic wound healing 121. For the same purpose, Li et al. also designed a GOX and hemin co-loaded G4-hydrogel as a cascade reaction container consuming endogenous glucose for wound treatment on diabetic mice 122.

Beside the glucose and O_2_ were used as substrates in infection-control cascade reactions, Br^-^ and H_2_O_2_ has also been used as substrates in the infection-control cascade reactions for preventing marine biofouling 73,74. For instance, V_2_O_5_ nanowires can transform Br^-^ and H_2_O_2_ into HOBr and ^1^O_2_ species due to its HPO-like activity. The formed ^1^O_2_ species exert strong antibacterial activity and thus prevents marine biofouling without toxicity to marine biota 73.

#### 3.2.2 Photo-enhanced nanozyme-based antibacterial therapy

As discussed above, enhancement of catalytic activity and binding capacity to bacteria are both crucial to realization of efficient antibacterial activity of nanozyme-based antibacterial agents. As known, nanozyme's activity can be improved by regulation of size, morphology, and compositions, surface modification, microenvironment adjustment, and application of external power 4, 8, 29, 41, 53, 55. In recent year, light regulation has proved to be a new and promising method to modulate the activity of nanozymes 40, 123-127. In the case of nanozymes, light regulation is one of the most attractive methods for tailoring the enzymatic activity owing to its excellent temporal and spatial precision compared to the existing approaches.

**Figure 4:**
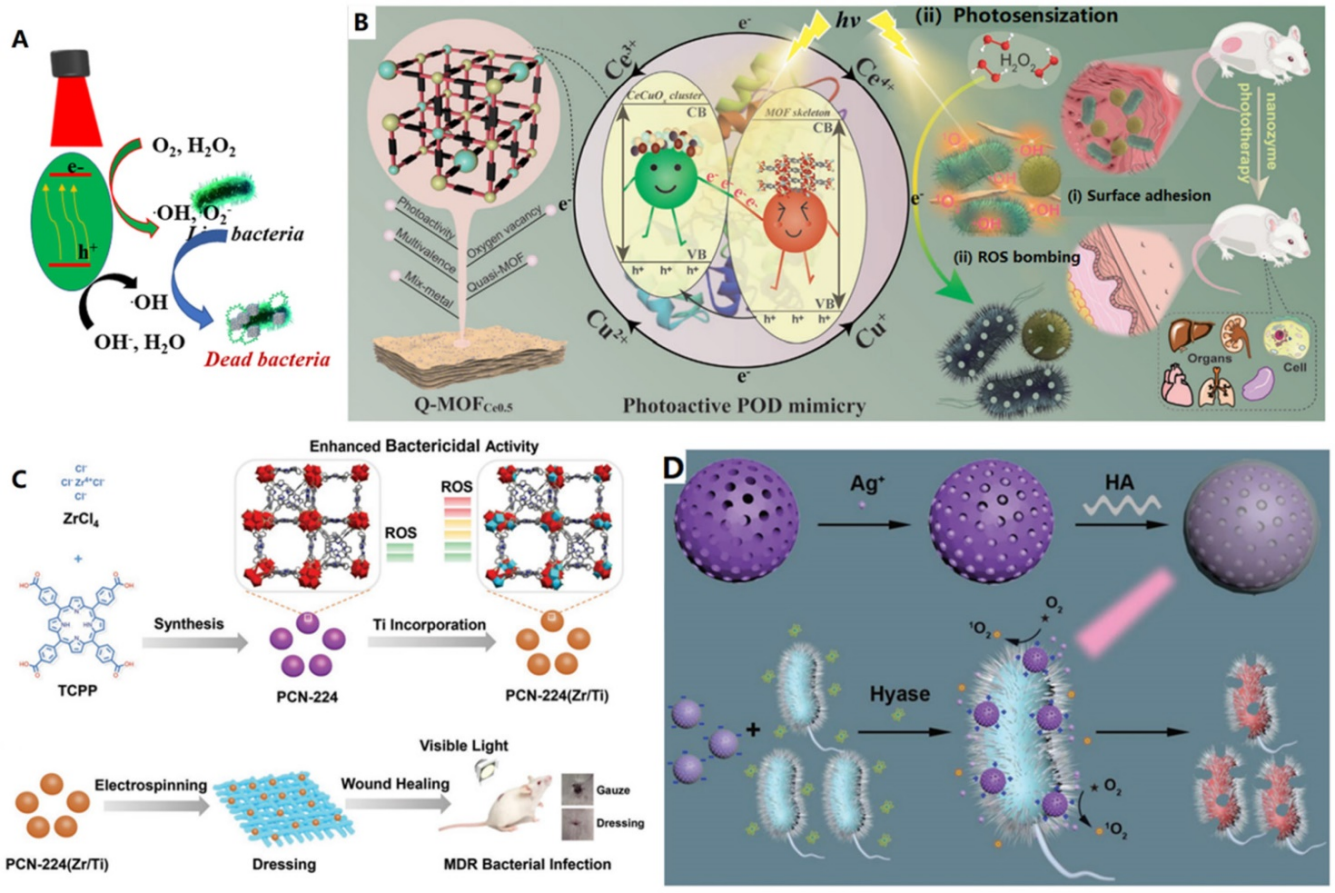
** A)** Schematic illustration of antibacterial mechanism of the photo-modulated nanozymes, adapted permission from ref. 40, Copyright 2019 the Royal Society of Chemistry. **B)** The catalytic disinfection mechanism proposed for the 2D bimetallic quasi-MOFCe0.5 nanozyme, adapted permission from ref. 134, Copyright 2022 the WILEY-VCH. **C)** Titanium incorporated Zr-porphyrinic metal-organic frameworks with enhanced antibacterial performance against multidrug-resistant pathogens, adapted permission from ref. 137, Copyright 2020 the WILEY-VCH. **D)** Silver-infused porphyrinic metal-organic framework as a surface-adaptive, on-demand nanoplatform for synergistic bacteria killing and wound disinfection, adapted permission from ref. 33, Copyright 2019 the WILEY-VCH.

Upon light excitation, some semiconductor-based nanomaterials can be initialized with light and generate ROS which can subsequently oxidize enzymatic substrates. Thus, inorganic nanoparticles used for photocatalysis and photosensitization were typically referred as light-driven oxidase mimetics or light-driven OXD-like nanozymes. In this regard, their introduction into the nanozyme community broadens the research range of the nanozymes 40. As shown in Figure 4A, under proper light irradiation, the semiconductor adsorbs light and initialized, the electrons in the valence band (VB) can be promoted to the conduction band (CB), leaving holes (h^+^) in VB. Consequently, O_2_ and H_2_O can be oxidized by the photogenerated electrons (e^-^) or holes to generate ROS such as hydroxyl (^.^OH) and superoxide (^.^O_2_^-^), which can be harvested for the oxidation of substrates, damage bacterial membrane and intracellular proteins and DNA, etc., thus photo-modulated nanozymes can kill bacterial efficiently 40. In the case of photosensitization process, the photosensitizer is firstly prompted to the excited singlet state and then activate the triplet state of oxygen to generate reactive oxygen species, which can be used for many photodynamic applications like antibacterial photodynamic therapies. Thus, photosensitizers were also regarded as photo-oxidative nanozymes 40.

Many semiconductor materials have been found to have photo-responsive enzyme-like activity 40,123-125. Moreover, the catalyst can be activated by a controllable light source, and free radicals can be produced to kill bacteria (Figure 4A) 129-138. Typically, many reports demonstrated that the enzyme-like activities of some nanozymes can be enhanced by light 55,91,135-138. For example, Huang et al., reported a bimetallic quasi-MOF (Q-MOFCe0.5) as a POD-like nano-mimetics for photodynamic antibacterial application both *in vitro* and *in vivo* under visible light irradiation (Figure 4B) 134. The representative Q-MOFCe0.5 was prepared by the MOF crystal engineering and controlled thermal transformation of corresponding pristine bimetallic MOF nanosheets. Furthermore, the Q-MOFCe0.5 is a hierarchical heterojunction-like 0D/2D composite in which the partial metal node-derived CeCuOx nanoclusters are uniformly distributed on the decarboxylated MOF skeleton. The heterojunction-like interface endowes the 2D decarboxylated MOF scaffold has isolated nodes-derived Ce-O-Cu sites with oxygen vacancy-coupled multivalent redox cycles and enhanced visible-light photosensitive energy band configuration. Therefore, the biomimetic quasi-MOFCe0.5 nanosheets exhibit enhanced photosensitive POD-like activity to generate sustained reactive oxygen species under visible light for efficient eradicating the surface-adhered bacteria *in vitro*. Furthermore, the biomimetic quasi-MOFCe0.5 nanosheets can also be used as a safe and on-demand POD-like antibacterial agent for healing potent disinfection of skin wounds *in vivo* 134. Liu et al. also reported a dumbbell-shaped Au@CeO_2_ hybrid nanozyme showed significantly enhanced POD-like activity upon NIR irradiation 135. By virtue of light-enhanced POD-like activity, Au@CeO_2_ can generate a large amount of ROS and kill efficiently a broad-spectral bacteria under 808 nm irradiation. Xi et al. reported that the POD-like activity of CuS NPs to generate ROS was enhanced under visible light irradiation 136. Compared to single bactericidal strategy, these kinds of synergetic modalities make the nanozymes excellent antibacterial candidates for accurate, on-demand and safer bactericidal therapies.

Up to now, most light-activated nanozymes were focused on the production of ROS. Except for the enhanced POD-like capability, some of the photo-trigged nanozymes can activate molecular oxygen directly to generate ROS for killing bacteria without usage of external toxic H_2_O_2_. A typical example is antibacterial photodynamic chemotherapy or named photocatalysis (Figure 4A). Among the various semiconductors, organic semiconductor photocatalytic materials have many advantages such as good biocompatibility, low cost, suitable band gap and diverse structural flexibility. Wang et al., constructed an all-organic photocatalytic heterostructure by combing C_3_N_4_ and perylene-3,4,9,10-tetracarboxylic diimide (PDINH) which exhibits enhanced photocatalytic efficiency because of the formation of a basal heterostructure. Thus, the nanocomposite could produce more reactive oxygen species to inactivate both Gram-negative and positive bacteria without obvious toxicity to normal tissue cells under simulated solar light (Figure 4B). Furthermore, this all-organic heterostructure show effective therapeutic effect for promoting wound healing *in vivo* 32.

For instance, as shown in Figure 4C, a bimetallic PCN-224(Zr/Ti) alone was used for photodynamic therapy (PDT) to heal MDR bacteria infected wounds. Under visible light, the obtained bimetallic PCN-224(Zr/Ti) was activated and generate high level of reactive oxygen species to effectively eliminate bacteria. Furthermore, a wound dressing with high biocompatibility and minimal cytotoxicity was prepared by loading the PCN-224(Zr/Ti) NPs onto lactic-co-glycolic acid nanofibers 137. Similarly, Qu constructed synergistic antimicrobial platform for the treatment of multi-drug resistant bacteria infected wound on mice model. Typically, the platform is powerful surface adaptive and on-demand. The platform was prepared by firstly loading Ag ions onto the photosensitive nMOFs and then coated with hyaluronic acid (HA) (Figure 4D) 33. Under light irradiation, the nanoparticles showed APDT ability for producing ROS to kill bacteria. Furthermore, in the presence of targeted bacteria, the Ag ions in PCN-224-Ag^+^ could be released quickly after the HA was degraded by the secreted hyaluronidase. Moreover, the produced positively charged nanoparticles have increased affinity to bacteria. Subsequently, the powerful surface adaptive and on-demand platform showed strong synergistic antibacterial effect on multi-drug resistant bacteria infected wound due to the synergistic performance of the released Ag ions and generated ROS. Wang et al. designed and synthesized a water stable V^4+^ based MOF material with regular mesoporous structure for the first time, and studied systematically the performance of the material in indoor humidity control and photocatalytic bacteriostasis 130. Results show that the nanomaterial has dual properties of water adsorption-desorption and photocatalysis bacteriostasis, and can be used for indoor humidity control, especially in the space shuttle cabin and submarine. The killing efficiency of BIT-66 to *E. coli* was 96% after exposure to visible light (400 nm < λ < 780 nm), which was much higher than that of MCM-41 (55%). ICP-OES and photocatalytic sterilization experiments verified that the excellent antibacterial performance of bit-66 mainly comes from the active oxygen radicals produced by photocatalysis, rather than the release of V^4+^.

Although antimicrobial photodynamic therapy (aPDT) or photocatalysis has shown great potentials in planktonic bacteria ablation treatment, it is difficult for the photosensitizers (PSs) to penetrate and diffuse inside the biofilm thus high concentration of PSs and dosage of light are desirable which are harmful for normal tissues. Moreover, therapeutic effects were limited because the microenvironment inside biofilm is hypoxic and oxygen rapidly deplete during PDT. To overcome this challenge, Qu et al., fabricated function-adaptive nanoplatform for efficient biofilms eradication 138. The pH/H_2_O_2_-responsive nanoplatform was prepared by encapsulating p-MOF dots (about 5 nm in-size) into human serum albumin-coated MnO_2_. It was noteworthy that the degradation of MnO_2_ triggered by the endogenous acidic microenvironment and over-pressed H_2_O_2_ could promote the control release of the p-MOF dots. The positively charged p-MOF dots could penetrate into biofilm effectively and attached tightly onto the negatively charged bacterial cell. Importantly, p-MOF dots in the biofilm could not only generate large amount of high ROS, but also can simultaneously generates O_2_ *in situ* and lessen hypoxia for biofilms. Thus, benefiting from above synergistic functions, the function-adaptive nanoplatform can effectively ablate bacterial biofilms *in vivo* without side effect to healthy tissues 138.

#### 3.2.3 Sonodynamic eradication of deep-seated bacterial infection

**Figure 5:**
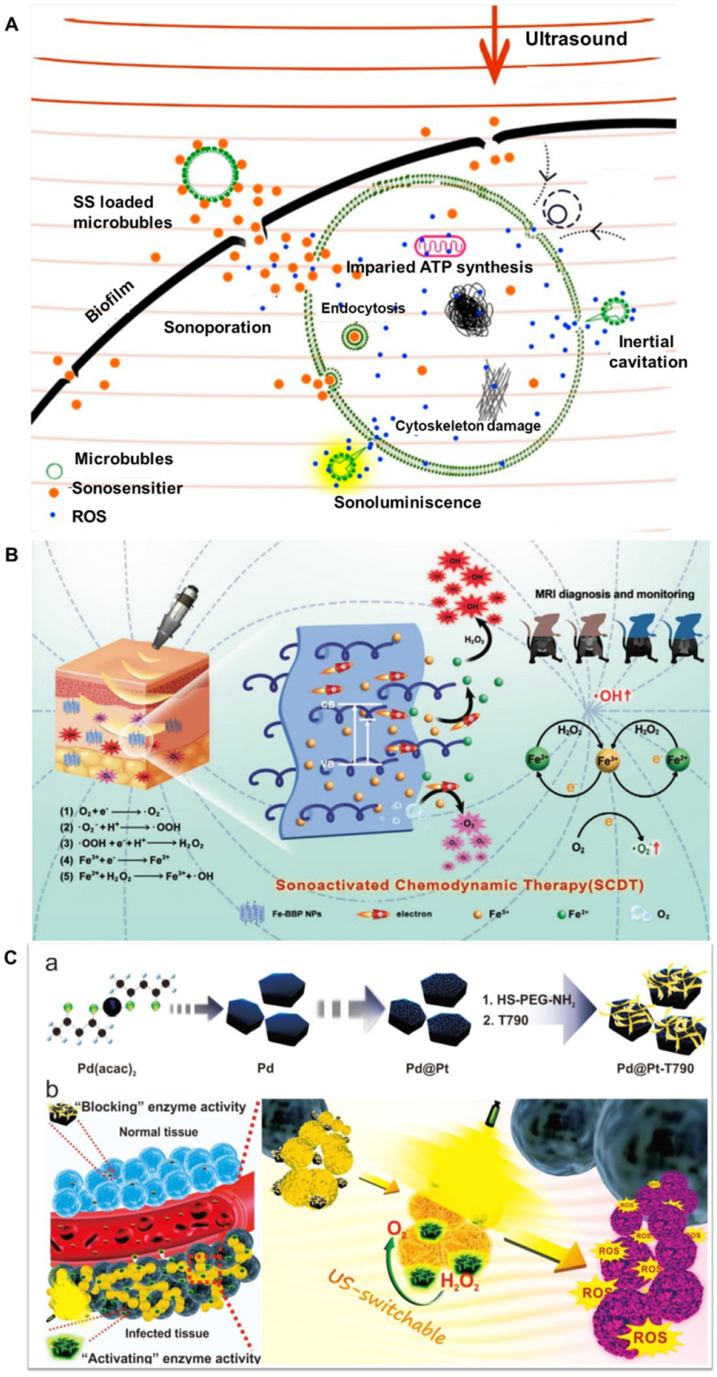
** A)** Proposed mechanisms of antimicrobial sonodynamic therapy, adapted permission from ref. 139, copyright@2021, the American Chemical Society. **B)** Schematic presentation of sonoactivated chemodynamic therapy (SCDT) against deep *MRSA* infection and the schedule of *MRSA* infection, systemic administration of Fe-BBP NPs, SCDT and monitoring *in vivo* adapted permission from ref.34, copyright@2020, the WILEY-VCH. **C)** Preparation of Pd@Pt-T790 nanoplatform and its US-switchable nanozyme catalytic oxygen generation-enhanced SDT of bacterial infection, adapted permission from ref. 140, copyright@2020, the American Chemical Society.

Light or ultrasound induced antibacterial approaches have been proved to be important alternatives against infectious microorganisms. Although antibacterial PDT for superficial tissues have achieved exciting developments, it is inefficient for deep-sited antibacterial infections because it is difficult for the light to penetrate inside the body. Alternatively, antibacterial sonodynamic therapy (SDT) is more attractive alternative than PDT for both superficial and deep-seated microbial infections (Figure 5A). These SDT methods have excellent tissue-penetrating capability and are highly biosafe because ultrasound is nonradioactive and has low tissue attenuation coefficient 139. However, their further clinical applications are severely limited due to the following disadvantages, such as the therapeutic efficacy of bacteria eradication is still low, and it is difficult to be localized in the infection site, therefore lead to undesired damage to surrounding normal tissues.

Up to now, great attention has been devoted to improve the efficiency of antibacterial SDT by developing new sonosensitizers with good bioavailability and high sonodynamic activity. For instance, Song et al. synthesized Fe-BBP NPs by grafting Fe^3+^ and branched PEI molecules on the surface of 2D BiOBr NPs via electrostatic interaction that was used as a theranostic platform for sonoactivated chemodynamic therapy (SCDT) against MDR bacterial infection (Figure 5B). Under low-frequency ultrasound, O_2_ would easily capture excited electrons to produce superoxide anions, while Fe^3+^ traps an interfacial charge to generate Fe^2+^, which can react with high concentrations of H_2_O_2_ in an inflammation microenvironment to form highly toxic ·OH. The proposed SCDT of Fe-BBP NPs can facilitate an accurate diagnosis and potent therapeutic effect for deep-seated MDR bacterial infection. Furthermore, MRI technique was used to *in situ* monitor the strong bactericidal effect of Fe-BBP NPs on mice with bacterial myositis 34.

As known, the microenvironment at the inflammation site is hypoxic, and the exposure of sonosensitizers to desired disease site is limited. Furthermore, the oxygen which is essential to the ROS generation depleted rapidly during SDT. Therefore, their practical applications are limited. Very recently, Sun et al. designed an US-switchable nanozyme (Pd@Pt-T790) to enhance the SDT efficacy by alleviating the hypoxia-associated barrier (Figure 5C). The catalase-like activity of Pd@Pt could be significantly blocked by the modification of T790 onto Pd@Pt. However, its activity could be effectively recovered upon US irradiation, which can catalyze the generation of O_2_ from endogenous H_2_O_2_. Due to such “blocking and activating” enzymatic activity, the US-switchable nanozyme could kill bacteria with minimal biotoxicity and side effects on normal tissues. As expected, the Pd@Pt-T790-based SDT nano-system can effectively eradicate *MRSA*-induced myositis because it could accumulated in infection sites without toxic side effect. In conclusion, the well-designed Pd@Pt-T790 with controllable activity could be used as efficient and precise US-switchable nanozyme to augment sonodynamic eradication of deep-seated bacterial infection 140.

Recently, effective combined diagnosis and therapy in the antibacterial field are high desirable. Thus, Wang et al., prepared multifunctional a Fe@UCNP-HMME nanoplatform for combined therapy and diagnosis. In this system, Fe_3_O_4_ cores and UCNPs shell can be used as MRI/UCL dual-modality imaging probes. Moreover, the HMME act as photosensitizer which can be irradiated by the NIR and visible light converted from NIR by the UCNPs for depth PDT and SDT, leading to the temporal induction of cytotoxicity and efficient killing of the deep-seated bacteria through damage of the cell wall structure 141.

### 3.3 Combination of more than one antibacterial mechanism

#### 3.3.1 Combination of metal ions and redoxase-like mimetics

Although silver nanoparticles (Ag NPs) are effective agents for killing drug-resistant bacteria, the reduction of the potential toxicity of Ag NPs is highly desirable but challenging. Therefore, the design of novel adaptive agents with lasting emission of Ag^+^ ions is necessary. Moreover, photocatalytic sterilization is more applicable. Under light irradiation, the photo-catalysts promoted the generation of sufficient ROS and kill the bacteria 32,129-131,142. More importantly, this green strategy does not induce antibiotic resistance and biotoxicity. Based on these advantages, Yang et al. designed a silver-based ternary composite ZnO/Ag/RGO as an efficient and green antibacterial agent for disinfection treatment. Under light irradiation, the silver nanoparticles in the composite could continuously release silver ions and the ZnO part could effectively generate ROS, respectively, resulting in synergistic antibacterial effect on *E. coli* and *S. aureus* 142. The continuously released silver ions from the silver nanoparticles can firmly adsorbed on the negatively charged bacterial cell membrane, thus interfering with bacterial DNA synthesis and making bacteria lose their ability of division and reproduction; ROS generated by photocatalysis such as ·O_2_^-^ and ·OH can decompose bacterial cell membrane and cause bacteria to rupture and die. Furthermore, the surface plasmon resonance effect of silver nanoparticles can not only broaden the absorption range of ZnO, but also accelerate the charge separation and effectively inhibit the recombination of photogenerated carriers. Later, Yu et al., prepared a novel antibacterial material by loading Ag NPs and Fe_3_O_4_ nanoparticles into chitin nano-fibrous microspheres 143. Ag-Fe_3_O_4_-NMs exhibit excellent antibacterial activity and enhanced wound healing ability attributing to synergistic effect of the released Ag^+^ and POD-like activity. Very recently, Huang et al. reported that a novel xerogel N-CD@ZnO could capture bacteria rapidly through electrostatic interaction and kill more than 99% of two types of bacteria under NIR light irradiation 144. Its high antibacterial efficiency is attributed to synergistic effect of continuous release of Ag^+^ and efficient generation of ROS by N-CD@ZnO. Furthermore, this xerogel could enhance the repair of bacteria-infected wound within 10 days.

In order to resolve the limitation of nanozymes in clinical medicine, Zhang et al., construct a multifunctional “antibiotic” (NH_2_-MIL-88B(Fe)-Ag). It not only can convert biological level H_2_O_2_ into highly active hydroxyl radicals but also can release Ag^+^ simultaneously, thus eliminate pathogenic bacteria completely 28. Furthermore, it could enhance the healing of the bacteria-infected wounds with negligible biotoxicity. In order to eliminate the biotoxicity and enhance efficiency of Ag-based antibacterial agent, Qu et al., fabricated an on-demand antimicrobial system with powerful surface-adaptive properties exhibiting a strong synergistic antimicrobial effect 33. In order to eliminate the toxic effect of Ag NPS, the photoactive MOFs were firstly loaded with Ag ions followed by coating negatively charged HA hyaluronic acid (HA) to prevent the leaking of Ag ions. The nanoplatform shows a strong synergistic antibacterial effect with good biocompatibility with non-targeted cells. However, the degradation of the HA on PCN-224-Ag-HA by the secreted HAase in the targeted bacteria leads to positively charged PCN-224-Ag^+^ with increased bacterial binding affinity. Benefiting from the synergistic effects of the released Ag^+^ and generated ROS by photocatalysis, the platform exhibits outstanding efficiency on killing MDR bacteria and accelerates the healing of the bacteria-infected wounds.

**Figure 6:**
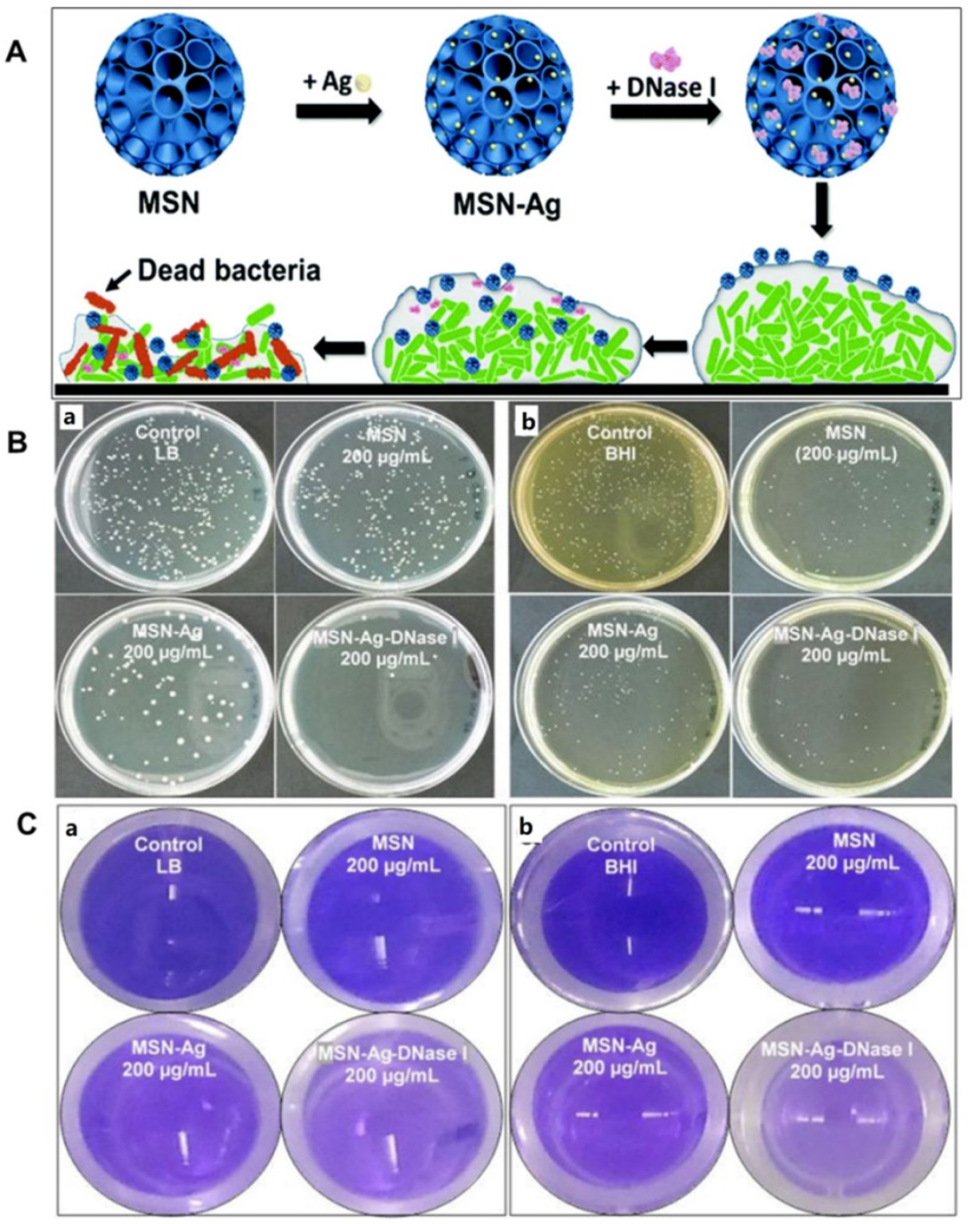
** A)** Schematic preparation of DNase I-loaded MSN-Ag nanocomposite for biofilm treatment. **B)** Antibacterial effects of nanoparticles towards bacterial biofilms of *E. coli* (a) and *S. mutans* (b). **C)** photograph of dispersal effect of DNase I loaded nanoparticles on bacterial biofilms of *E. coli* (a) and *S. mutans* (b) visualized with crystal violet staining, adapted permission from ref. 35, copyright@2020 the Royal Society of Chemistry.

Beside the “intrinsic antimicrobial-resistance”, “infectious biofilm” is another problem that hampers infection control. Most infections are caused by bacteria in their adhering, biofilm-mode of growth 3. The matrix of EPS act as barriers to protect the bacteria. Therefore, bacteria in the film are usually difficult to kill. It has been proved that deoxyribonuclease I (DNase I) can damage the biofilm matrix by breaking down the extracellular DNA of EPS. Furthermore, Ag nanoparticles could kill the bacteria though ROS generation and other binding with biomolecules inside the cell. In order to use both functions of Ag and DNase I, Xu et al. load DNase I into MSNs with large cone-shaped pores doped with silver nanoparticle for the biofilm treatment (Figure 6A). Due to the high dispersion of Ag NPs and large amount of DNase I in the mesoporous silica, the nano-formulation showed enhanced antibacterial effects for both Gram-negative and Gram-positive biofilms (Figure 6B and 6C) 35. Beside Ag^+^, Zn^2+^ also has good antibacterial effect. Thus, Wang et al. developed a triple-synergistic strategy by combing the glucose-induced cascade reaction and leaching of Zn^2+^for efficient bacterial eradication. Due to the ROS production and released Zn^2+^, the triple-synergistic strategy achieved enhanced overall bacterial eradication at a low dosage for both *S. aureus* and *E. coli* 119.

#### 3.3.2 Dual enzyme-like nanozymes as synergetic antibacterial systems

More than 80% of the human bacterial infections are induced by bacterial biofilms, which are hard to eliminate because of its EPS connected by eDNA. Therefore, eDNA plays vital function in maintenance of biofilm structure. DNase can hydrolyze eDNA and thus disassemble biofilms. However, DNase is expensive and instable. To circumvent these issues, Qu et al. designed a series of dual enzyme-mimetic nanozymes to combat biofilms. The dual enzyme-mimetic nanozymes used grafted Ce(IV) complexes as deoxyribonuclease-like mimic to hydrolyze eDNA and MOF doped with Au as POD-like mimetic to generate ROS. Benefiting from the synergetic performance of the above dual enzyme-like activities, the nanozymes could disrupt biofilms and combat bacteria inside 69.

#### 3.3.3 Synergy antibacterial system combining photothermal effect with redoxase-like mimetics

Recently, NIR laser-induced hyperthermia or photothermal therapy (PTT) based on such light absorbing nanomaterials has become a promising and non-invasive strategy to eradicate pathogenic bacteria 147-154. It can kill microorganisms through a physical sterilization method, in which way the proteins such as enzymes, cell membrane can be effectively destroyed by the hyperthermia effects induced by converting light into local heat. Unfortunately, their clinical application is still a challenge because it is not biosafe with long-term exposure to NIR laser. The construction of synergistic dual phototherapy nanoplatform by combing POD-like nanozymes with photothermal function is expected to facilitate more satisfactory therapeutic effects than single antibacterial mode. Typically, the produced ·OH by nanozymes can damage the cell membrane and endow it with improved permeability and sensitivity to heat, thus hasten bacterial treatment and eliminate the side effects of PTT 147-155. Moreover, the nanozyme could eliminate inflammatory and promote wound healing without obvious cytotoxicity to healthy mammalian cells. For the same purpose, some nanozyme can generate hyperthermia under light irradiation which can enhance both their POD-like catalytic activity and the membrane permeability, thus significantly improving their antibacterial disinfection ability 147-155.

In this regard, recently, Yin et al. prepared a MoS2 nanoflowers-based synergistic antibacterial system for effective bacteria elimination *in vitro* and wound disinfection *in vivo* by integrating its peroxidase-like activity and NIR induced hyperthermia 147. The PEG-functionalized nanostructured MoS_2_ could efficiently promote the conversion of H_2_O_2_ to more toxic hydroxyl radicals (·OH) for killing bacteria. Furthermore, NIR induced hyperthermia further accelerate the death of *E. coli* and *B. subtilis.* Therefore, this synergetic antibacterial system could kill bacteria rapidly with negligible toxicity using low concentrated H_2_O_2_. Pan et al. also prepared a photothermal and nanozyme integrated synergetic nanoplatform by combing reduced graphene oxide(r-GO) and iron oxide nanoparticles (IONP) for combating *MRSA*. The nanocomposite can not only kill bacteria physically by inducing hyperthermia under exposure to near infrared (NIR) energy, but also chemically damage* MRSA* by degrading H_2_O_2_ into highly reactive •OH radicals. Furthermore, the synergetic nanoplatform could effectively kill the bacteria in subcutaneous abscesses and promote wound healing 148. Recently, Liu et al. used a dumbbell-shaped Au@CeO_2_ nanozyme as a broad-spectrum antibacterial agent for efficient bacteria disinfection under NIR irradiation. Its high antibacterial efficiency was attributed to the synergetic effects of plasmonic-promoted POD-like activity and NIR induced hyperthermia 135.

In order to enhance the antibacterial properties and reduce biological toxicity of N-doped porous carbon, Wang et al., doped copper single-atoms into N-doped porous carbon (Cu SASs/NPC) by a simple strategy 149. The Cu-doped NPC exhibits improved catalytic performance of nanozymes and hyperthermia under NIR light, depleting GSH in bacteria and generating plenty of hydroxyl radicals (•OH) to kill bacteria. Benefiting from synergistic antibacterial effects, the Cu-doped NPC can disinfect *E. coli* and *MRSA* with almost 100% efficiency and promote the *MRSA*-infected mouse wounds healing.

Zhang et al. also reported a gold-platinum nanodots-based combined therapeutic strategies for bacterial infection and bacterial-infected would healing 150. Xu reported a WS_2_QDs-Van@liposome-based photo-controlled antibacterial nanoplatform with both photothermal and nanozyme properties. In this platform, POD-like WS_2_ QDs and vancomycin were loaded in thermal-sensitive liposome. Under NIR irradiation, the thermal-sensitive liposomes are disrupted due to the photothermal property of WS_2_QDs. Thus, the antibiotics are released precisely and the antibiotic dosage are greatly reduced. Importantly, a synergistic antibacterial effect could be achieved by combing the antibiotic vancomycin with the hyperthermia effect and POD-like and OXD-like activities of WS_2_QDs 36.

Biofilm is a three-dimensional structure formed by microorganisms attached to biological or non-biological surfaces. Compared with planktonic-bacteria, it is more challenge to eliminate biofilm-related infections. To overcome the major challenge for removal and sterilization of bacterial biofilms in root canals, Cao et al., proposed an efficient strategy for disinfecting biofilm-protecting bacteria by integrating photothermal and POD-like catalytic activity of nanocomposites (Au@Cu_2_-xS) 154. The nanocomposite could convert H_2_O_2_ at physiological relevant concentrations to a large amount of •OH to decompose proteins and polysaccharides-the main components of biofilm. Furthermore, the nanocomposite could generate imperative hyperthermia to denaturalize the heat shock proteins. Benefiting from synergistic effect of POD-like activity and photothermal properties, the Au@Cu_2_-xS nanocomposite-based synergistic antibacterial system achieve high sterilization efficiency against two types of bacteria and is a promising approach to combat bacteria and biofilm in root canals 154. Zhang et al. reported that an oxygen-vacancy molybdenum trioxide nanodots (MoO_3_-x NDs) was used as an efficient and safe bacteriostatic combining its photodynamic, photothermal, and peroxidase-like enzymatic activities under NIR irradiation (808 nm) 155. Furthermore, it can effectively enhance the healing of *MRSA*-infected wound in living systems. Very recently, Liu et al. also reported that the surface roughness of carbon-iron oxide nanoparticles plays key roles in efficient bacteria disinfections. Benefiting from synergistic effects of rough surface and the photothermal property of carbon nano-shells as well as the POD-like activity of iron oxide nanoparticles, it could realize more effective antibacterial therapy under low-energy NIR-II light irradiation with excellent biocompatibility 30.

**Figure 7:**
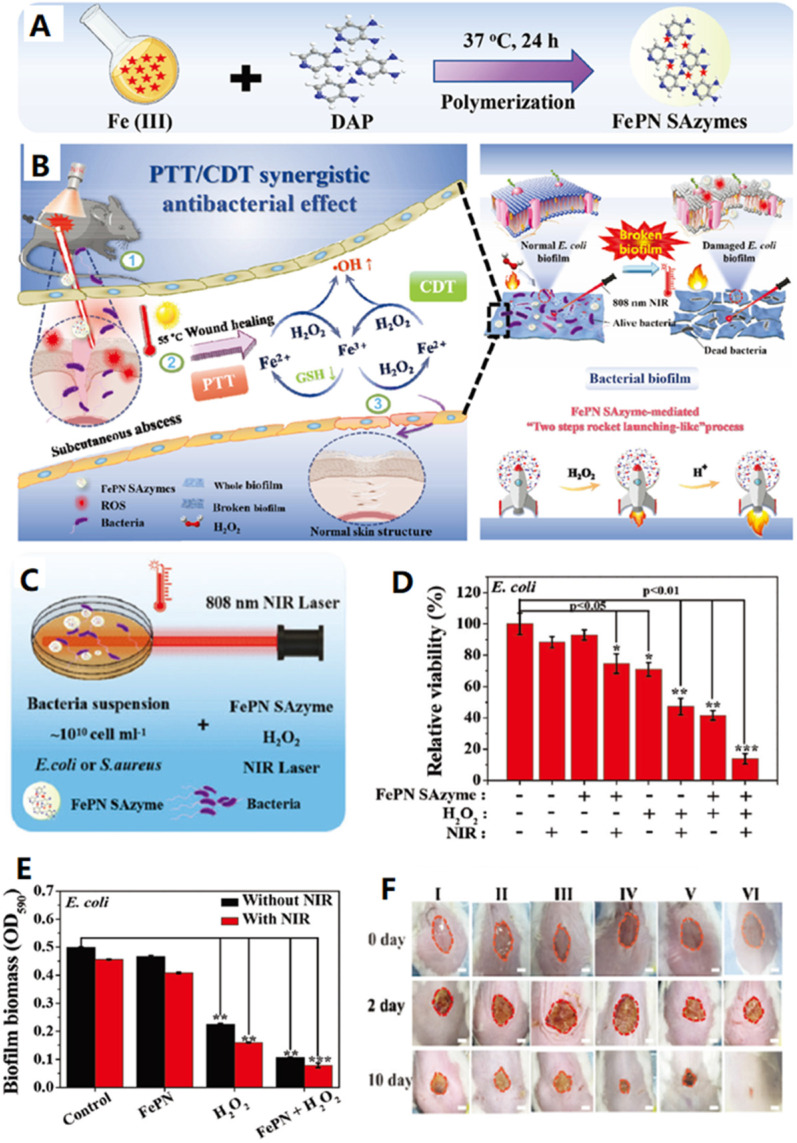
**A)** Schematic procedure of the synthesis of FePN SAzyme. **B)** Schematic illustration of the mechanism of the synergetic PTT and CDT bacteria-infected wound therapy using FePN SAzyme as a biofilm-microenvironment-activated single-atom nanozyme. **C)** Schematic illustrates NIR irradiation-enhanced ROS generation by peroxidase-like activity of FePN SAzyme for antibacterial treatment. **D)** Relative viability of *E. coli* incubated with FePN SAzyme with or without H_2_O_2_ under 808 nm laser irradiation. **E)** Biofilm biomass of *E. coli* based on the OD_590_ value for assessing changes in after different treatments. **F)** Photographs of wound on the mice at different time points after different treatments. I) PBS, II) FePN SAzyme (100 µg/mL), III), H_2_O_2_ (100×10^-6^ M, 5 µL). IV) FePN SAzyme+808 nm (1 W/cm^2^, 10 min). V) FePN SAzyme+H_2_O_2_. VI) FePN SAzyme+808 nm+H_2_O_2_. Adapted permission from ref. 156, copyright@2021 the WILEY-VCH.

Compared to healthy tissues, biofilm microenvironment is hypoxia, acidic, and is rich of H_2_O_2_ and glutathione. Thus, it is more challenge to eliminate biofilm-related infections. In this regard, the exploration of biofilm microenvironment-activated reactions for killing bacteria is an artful way to eradicate bacterial biofilm infections 156,157. Very recently, Yong's group prepared a Fe-doped polydiaminopyridine nanofusiform-mediated single-atom nanozyme (FePN SAzyme) through a facile one-pot, surfactant-free aqueous polymerization strategy (Figure 7A) and used as a biofilm microenvironment-activated nanozyme-based activatable nanoplatform for healing bacteria-infected wound (Figure 7B) 156. As shown in Figure 7C and D, FePN SAzyme/H_2_O_2_/808 nm laser system show the highest antibacterial activity for inhibiting the growth of *E. coli* bacteria in the presence of H_2_O_2_ under NIR light irradiation, revealing its dominant POD-like antibacterial performance effectively enhanced through combining the photothermal effect. Furthermore, the group of FePN SAzyme/H_2_O_2_/808 nm laser system gives the best antibacterial activity for inhibiting formation of the *E. coli* bacteria-induced biofilm (Figure 7E), because the FePN SAzyme with single Fe atom active center is positively charged and could penetrate and accumulate in biofilms due to electrostatic interactions. Moreover, the high concentration of H_2_O_2_ in the biofilm microenvironment promotes its PTT effect which was further accelerated by the mild acidic inflammatory environment. Meanwhile, the nanozyme has enhanced chemo-dynamic effect by promoting the oxidation of biofilm-overexpressed GSH with H_2_O_2_ to produce hydroxyl radicals. Additionally, the biofilm-overexpressed H_2_O_2_ can be catalytically converted into O_2_ to eliminate the hypoxia of biofilm. In these regards, the nanoplatform provides significantly synergistic therapy for biofilm-related infections. Encouraged by the above results, the group further confirmed the feasibility of FePN SAzyme for proliferation inhibition of bacteria and potential wound healing efficacy *in vivo* (Figure 7F). More importantly, the nanoplatform is safe for the healthy tissue because it is simultaneously switchable by the internal and external stimuli. In summary, this nanoplatform has great potential in biofilm-specific anti-infection therapy.

### 3.4 Selective or targeted disinfections

Nanozyme can kill broad-spectrum of Gram-negative and Gram-positive bacteria. However, it is more significant to selectively discriminate and precisely eliminate pathogenic bacteria. Normally, nanozymes can selectively discriminate and eliminate bacteria by taking advantage of electrostatic and hydrophobic interactions with the target bacteria, molecular recognition, microenvironment response and others.

#### 3.4.1 Selective discrimination of bacteria based on electrostatic and hydrophobic interactions

As known, the surface charge and hydrophobicity of the cell wall components for Gram-positive and Gram-negative bacteria are significant different. Normally, Gram-positive bacteria has negatively charged surface containing teichoic acids and lipoteichoic acids with a thicker peptidoglycan layer. However, Gram-negative bacteria is composed of a bilayer membrane with abundant negatively charged lipopolysaccharides in the outer membrane. Furthermore, there are cross-linked lipopolysaccharides by divalent cations in Gram-negative bacteria, thus inhibiting the interaction of hydrophobic molecules with phospholipid 158. In this regard, Zhao et al. prepared hydrophobic CNDs for selectively discrimination and elimination for obligate anaerobes. The hydrophobic CNDs can selectively insert into the cell membrane of Gram-positive bacteria through hydrophobic interaction, and adsorb NIR to generate powerful ROS on the targeted bacteria surface under light excitation, inactivating the Gram-positive bacteria by damaging the cell membranes. It is worth to note that CNDs could kill 99.99% of *MRSA* under 660 nm light irradiation for 5 min, but is not active for killing *E. coli*. Moreover, CNDs could heal the wounds infected by *MRSA* faster than the control group without CNDs 159.

Chen et al. reported that antibacterial activities of noble metal-based NPs could be regulated by simply tuning their exposed facets. By investigating the enzyme-like activities and antibacterial performance of Pd NPS with different exposed facets, they found that Pd nanozymes with different crystal surfaces can selectively eliminate Gram-positive or Gram-negative bacteria 53. There is an important correlation between the surface morphology of Pd nanocrystals and facet-dependent oxidase and POD-like activities. Furthermore, the antibacterial efficiency against Gram-positive bacteria of Pd nanocrystals shows the same trend with their enzyme-like activity for generating ROS. However, in the case of Gram-negative bacteria, a reverse trend is observed. This is because the ROS is not sensitive to Gram-negative bacteria.

**Figure 8:**
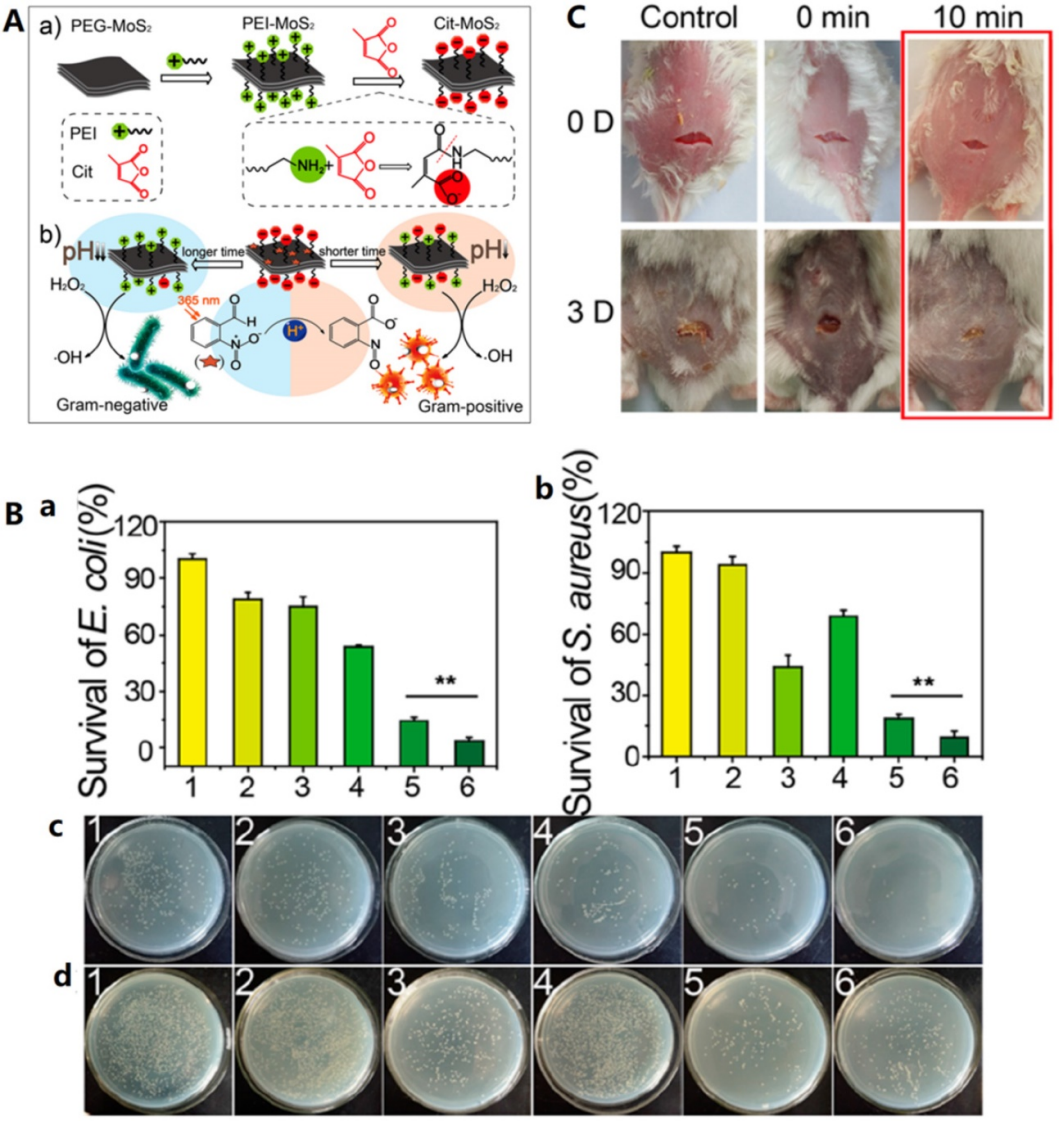
** A)** Schematic illustration of gram-selective antimicrobial performance of Cit-MoS_2_ (III) based-nanozyme under light-modulation. **B)** Antibacterial activity of the systems by a growth-inhibition assay in liquid LB medium and a plate counting method. Numbers of the surviving bacteria *for E. coli* (a) and *S. aureus* (b) by plate counting method, respectively. (c, d) Represent plate counting images of (c) *E. coli* and (d) *S. aureus* after different treatments. 1) PBS; 2) 2-NBA (40 min, irradiation time); 3) Cit-MoS_2_ (III); 4) H_2_O_2_; 5) 2-NBA + Cit-MoS_2_ (III) (40 min, irradiation time); 6) H_2_O_2_ + 2-NBA + Cit-MoS_2_ (III) (40 min, irradiation time), respectively. [Cit-MoS_2_ (III)] = 500 µg/mL, [H_2_O_2_] = 10 mM, [2-NBA] = 600 µM, λ = 365 nm. **C)** Photographs of *S. aureus* infected wounds on the mice after treatment with Cit-MoS_2_ (III) based-nanozyme under different irradiation time, respectively; Adapted permission from ref. 37, copyright@2018, the American Chemical Society.

In addition to the different selectivity of the material itself to bacteria, the selective antibacterial activity of nanozyme can also be realized through combination strategy. Qu groups also developed an intelligent strain-selective antimicrobial system using photoacid modified charge-tunable MoS_2_ 37. The surface-charge can be modulated from negative to positive by simply changing the light irradiation time. Because of the difference of cell structures of bacterial strains, charge-selective antibacterial activity is achievable by the photo-regulated charge-tunable nanozyme. As shown in Figure 8A, under light, the photoacid molecule 2-nitrobenzaldehyde is converted into 2-nitrobenzoic acid, which has two effects: first, the surface charge of polymer molecule modified molybdenum sulfide in pH response is converted from negative charge to positive charge, which is conducive to the selective combination of materials with bacteria; At the same time, the solution pH changes from neutral to acid, which activates the peroxidase activity of molybdenum sulfide. The above two effects have achieved highly efficient and selective antibacterial performance of light regulation 37. The antibacterial ability of the system was evaluated by a growth-inhibition assay in liquid LB medium and a plate counting method, the results are summarized in Figure 8B. Compared to other five groups, the group treated with 2-NBA mixed Cit-MoS_2_ (III) in the presence of H_2_O_2_ (irradiation with 365 nm for 40 min) exhibited the best killing ability toward *E. coli*, confirming the synergistic antibacterial performance between the increased positive charge and enhanced peroxidase-like activity. In addition, similar trend was obtained for *S. aureus.* The group further investigate the pH environments and the irradiation time effect on the antibacterial selectivity. At weakly acidic conditions, Cit-MoS_2_ (III) selectively inhibited the growth of *E. coli*. However, under near neutral pH environment, the system selectively suppressed the growth of *S. aureus*. Moreover, after long-time irradiation, the surface of Cit-MoS_2_ (III) is positive, thus is more effective in inhibiting Gram-negative *E. coli*. Finally, bacteria infected wound on mice model was used to evaluate the Gram-selective antibacterial activity of the designed light-activated antimicrobial system *in vivo*. As shown in Figure 8C, excellent therapy for *S. aureus* infected wound on mice was achieved with 10 min of irradiation. Recently, an *in vivo* activatable pH-responsive PtCo@Graphene nanozyme was used in strain-selective antimicrobial system for selective treatment of *Helicobacter pylori* (*H. pylori*) infection. The nanozyme is stable and active in acidic gastric milieu and exhibit targeting bacterial killing ability toward *H. pylori* without side effect on normal tissues 160.

#### 3.4.2 Selective discrimination of bacteria based on molecular recognition

Many reports proved that Gram-positive or Gram-negative bacteria can be selectively identified and eliminated by conjugating targeting ligands such as antibiotics with phototherapeutic agents 23. Vancomycin (van) is a narrow-spectrum antibiotic that has been widely used to combat infection arising from gram-positive bacteria such as *S. aureus*. It is a glycopeptide antibiotic which can bind to a specific C-terminal sequence in the peptidoglycan pentapeptide in the bacterial cell wall. Therefore, Van molecule has the bacteria-targeting ability for the gram-positive bacteria 161-163. Based on its targeting ability, many vancomycin-functionalized nanomaterials were used for targeted antibacterial applications. For example, Azzam et al., fabricated a novel furry amino magnetic poly-L-ornithine (PLO)/amine-poly(ethylene glycol) (PEG)-COOH/vancomycin (VCM) (AM-PPV) nanospheres with high-loading VCM to track and capture pathogens. The nanospheres could efficiently capture and detect *B. cereus* with high accuracy and selectivity at low levels due to their relatively rough surface and the bacteria-targeting ability of Van molecule 161. Furthermore, a vancomycin-functionalized porphyrinic MOFs (PCN-224) was used for targeted antibacterial application. Due to the integrated merits of the PDT feature of PCN-224 and the bacteria-targeting ability of Van molecule, the multifunctional antibacterial agent not only selectively targeted the Gram-positive bacteria but also exhibited enhanced ability for combating bacteria 162.

### 3.5 Nanozymes for suppressing intracellular bacteria

Although antibiotics are most commonly used agents for antibacterial therapy, the elimination of intracellular bacteria is still challengeable because the intracellular bacteria can be protected by the infected cells by deactivating antibiotics and blocking their antibacterial action. Therefore, it is highly desirable to develop novel and efficient antibiotic-alternatives to eliminate the intracellular intracellular antibiotic-resistant bacteria thus prevent the recurrence of infection and chronic inflammation 164-168.

*Serotype Salmonella Enteritidis (S. Enteritidis)* is a facultative intracellular pathogen and can cause intracellular infections 169. Furthermore, it is highly resistant to multiple antibiotics. Thus, the development of novel and environmentally-benign antibiotic-alternatives to eliminate intracellular infectious caused by intracellular *S. Enteritidis* is important for protecting both animal and human health. In this regard, Shi et al. developed an IONzymes for enhanced autophagic elimination of intracellular* S. Enteritidis* in LMH cells. Results demonstrated that the IONzymes co-localized with* S. Enteritidis* in autophagosomes could promote the autophagic activities and generate ROS in the acidic microenvironment, thus lead to enhanced antibacterial performance. Typically, the bacteria in the livers of the *S. Enteritidis*-infected chickens could be reduced greatly by oral administration of IONzymes. Moreover, by using “non-target high-throughput omics” technologies, the authors confirmed the hepatic oxidation-reduction and autophagy in *S. Enteritidis* infected chickens are ascribed to the effects of IONzymes on genes. In summary, IONzymes are effective, safe and cost-effective antibiotic alternative for the clinical therapy of the *S. Enteritidis* associated intracellular infections 170.

Antibacterial photodynamic therapy (aPDT) has proved to be promising method for the elimination of intracellular bacteria by converting nontoxic oxygen to toxic ROS under light irradiation. In this regard, Liu et al. prepared a ZnPc@ZIF-90-M system that can effectively eliminate both planktonic bacteria and macrophage-entrapped antibiotic-resistant bacteria. In this system, photosensitizers of molecular ZnPc loaded in the nanocage of ZIF-90 are monodispersed and effective for ROS generation and antibiotic-resistant bacteria elimination. Furthermore, the coating of a mannosylated shell endow the ZnPc@ZIF-90-M with targeting and internalizing ability with macrophages. In summary, the MOF-based nanoplatform provide a novel direction for designing targeted system for the elimination of stealthy antibiotic-resistant bacteria 171.

### 3.6 Multifunctional platforms for “detection and inhibition” of bacteria

**Figure 9:**
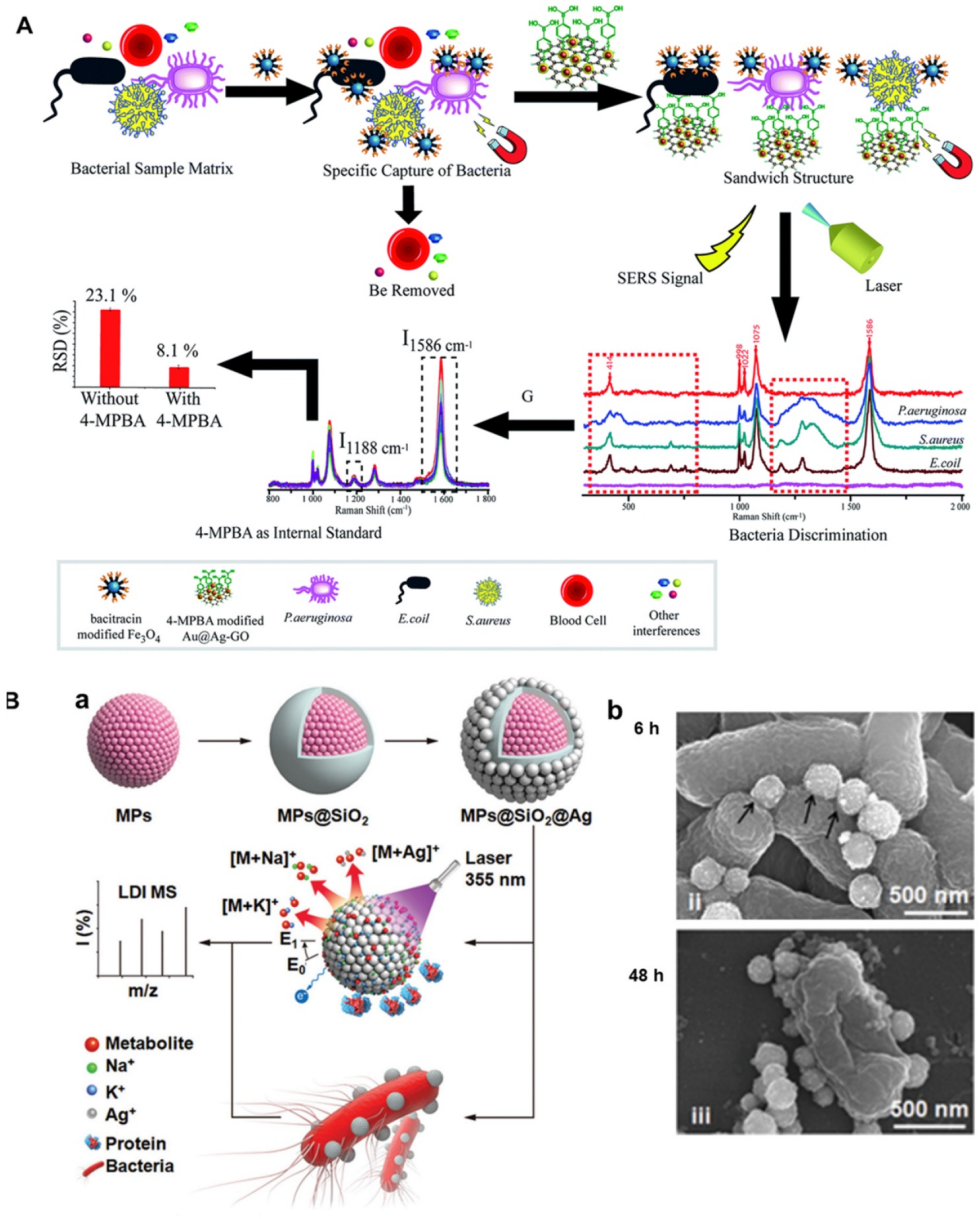
** A)** A multifunctional nanoplatform for isolation, discrimination and killing of multiple bacteria in whole blood, adapted permission from ref. 24, copyright@2018, the Royal Society of Chemistry. **B)** Schematic diagrams of a dual-functional silver platform for detection and inhibition of bacteria (a) and SEM images of bacteria treated for 6 h and 48 h with dual-functional silver platform. Adapted permission from ref. 25, copyright@2019, the WILEY-VCH.

Developing a dual- or multi-functional platform for “detection and inhibition” of bacteria is in demand but challenging, because bacterial infection has become a major threat to global public health and the use of targeted antibiotics for treatment is often used clinically. Traditional detection methods, such as standard plate colony count, immunoassay and PCR, are complex and time-consuming, most of which require expensive special equipment. Surface enhanced Raman scattering (SERS) may realize the quick, sensitive and effective detection of bacteria. This method can provide sharp, specific fingerprint spectra of the bacteria, thus different kinds of bacteria can be discriminated among from a mixed sample matrix. Furthermore, antimicrobial peptides are attractive bacteria capture due to its high stability and low cost. In view of the above problems, Yuan et al. developed a new biosensor which can simultaneously isolate and detect a variety of bacterial pathogens 24. As shown in Figure 9A, in this biosensor, the Fe_3_O_4_ magnetic nanoparticles modified with antibacterial peptide are used as “capture” probes to selectively separate and magnetically enrich bacteria. 4-MPBA modified gold- silver-graphene oxide (Au@Ag-GO) nanocomposites are used as SERS tags. 4-MPBA can be used as both biomacromolecule recognition and Raman signal molecule for internal standard correction of SERS intensity. Three model pathogens were successfully isolated and detected by this new biosensor. The minimum detection limit of each strain was low to 10 CFU/mL. Moreover, Fe_3_O_4_ magnetic nanoparticles modified by antimicrobial peptides have high antibacterial activity and low cellular cytotoxicity, which can be used as antimicrobial agents in long-term blood storage. Finally, the new method is applied to the detection of blood samples from clinical patients. Thirty-nine patients' real blood samples were detected and more than 97% of which were effectively isolated and detected. This new ternary biosensor with the three in one function of “isolation, discrimination and killing” of pathogenic bacteria is expected to have great application potential in clinical diagnosis, blood transfusion safety assurance and other fields.

Metabolic analysis is more distal than conventional proteomic and genomic approaches for detection of bacteria in real biological samples. But it is difficult to be applied in bacterial diagnostics because of high complexity of biosystems and low abundance of metabolites. Recently, Vedarethinam et al. also built a multi-functional platform for both detection and kill bacteria 25. As shown in Figure 9B(a), bacterial metabolites in complex biological liquids can be analyzed by laser desorption ionization mass spectrometry to achieve clinical grade monitoring of serum bacterial infection; Moreover, the surface of magnetic silver with rough surface is easy to be attached on the bacterial surface, which is favorable for the release of silver ions and killing bacteria. Under the cooperation actions of nanoparticles and silver ions, a long-term bacteriostatic effect can be obtained by destroying the bacterial membrane and leading to the disorder of protein synthesis 9B(b). This work provides a novel strategy for the design of multi-functional platforms for “detection and kill bacteria” of pathogenic bacteria. With such multi-functional platform, accurate, rapid and high-throughput detections of bacterial metabolic markers can be realized. Meanwhile, bacteriostatic mechanism can be better understood and more efficient antibacterial effects can be achieved through monitoring metabolic changes.

Electrochemical methods have proven to be one of the most promising strategies for rapid bacteria detection method because of their advantages such as real-time detection, high sensitivity and rapid conversion of chemical signals into electrical signals. Therefore, Wu et al. demonstrated a three-in-one impedance-based electrode sensor which could efficiently capture, rapidly kill and ultra-sensitively detect bacteria 26. The impedance-based sensor was constructed by decorating Zn-CuO nanoparticles with rough surface and graphene oxide nanosheets on a Ni porous electrode. The integrated platform with burr-like surface could capture 70-80% of bacteria at a concentration of 50 CFU/mL in 20 min, and completely kill bacteria in 30 min. Furthermore, its quantitative detection sensitivity for bacteria is very high and is also effective in a biological sample. Similarly, Wang et al. also constructed an “on-demand” multifunctional magnetic electrode for accurate diagnosis and selectively combating *S. aureus*. The antibody modified on the magnetic glassy carbon electrode can specifically capture *S. aureus* in solution. Therefore, the concentration of *S. aureus* solution can be detected with high detection sensitivity due to the good relevance between the concentration of *S. aureus* and peak current. Furthermore, benefiting from the controlled released van and good biocompatibility, it could combat *S. aureus* effectively in the whole blood 21.

## 4. Summary and outlook

In this review, we summarized the recent progress of nanomaterials-based multimodal synergistic therapies in combating bacterial infections, including the combination of Ag ions and ROS, photothermal and ROS production, and cascade reactions et al. The current state of the art nanozyme-based multimodal synergistic therapies exhibits great potential in treating localized skin infections including wound biofilms in the coming future. But there are still several problems that need to be addressed.

- At present, the antibacterial spectrum for most nanozymes-based antibacterial systems are mainly investigated using *E. coli*, *S. aureus* and *Pseudomonas aeruginosa* (*P. aeruginosa*) as model bacteria, their bactericidal effects on other pathogenic bacteria are not clear. Furthermore, whether the nanomaterials-based multimodal synergistic antibacterial systems could induce drug resistance should be more concerned. The nanozyme mediated ROS attack is random and less selective. Therefore, the development of new strategies to realize the antibacterial specificity of nanozyme is high desirable. Although nanozyme has good bactericidal activity, its current investigations are mostly limited to in-vitro or skin surface, and little research is on in-vivo application. It is highly desirable to evaluate their bactericidal efficiency and biosafety of nanozyme *in vivo*.
- Although many efforts have been devoted to the antimicrobial SDT, there are still challenges to be overcome for clinical purposes. Such as the hypersensitivity to light post-therapy because most of the sonosensitizers are photosensitive. Therefore, the development of novel alternatives that are less photosensitive under the exposure of light is one of the attractive strategies to improve the biosafety of the antibacterial SDT.
- Recent studies confirmed that there are bacteria in the tumor and the immune cells. The intracellular bacteria could produce some toxin to make the cells are in an inflammatory state and promote cancer. However, the elimination of intracellular bacteria is still challengeable because the intracellular bacteria can be protected by the infected cells by deactivating antibiotics and blocking their antibacterial action. Therefore, the development of smart and targeting nanozyme-based platforms that could kill both bacteria and cancer simultaneously is highly desirable in the clinical treatment.
- The construction of multi-functional platform of “isolation, detection and killing” for collecting pathogens is one of the most important directions for future development. Furthermore, nanozymes not only could be used for bacterial sterilization by regulating ROS, but also for many other diseases which related to ROS level, such as tumor occurrence, cardiovascular disease and neural aging.
- Recently, nanozymes have been considered to be a new but rapidly developing cross-fields of nanobiology, and their antibacterial applications is just in beginning stage. We believe that with the deepening of research, the applications of nanozymes could be transferred from basic research to clinical transformation as new antibacterial agents. They can be used to prevent and control infection of various diseases, to solve the problem of antibiotic resistance and abuse, and improve the quality of human health life.

## Figures and Tables

**Table 1:** Summary of synergistic antibacterial systems based on nanozymes with rough surface for antibacterial disinfections

Strategies	Nanomaterials	Enzyme-like activities	Antibacterial mechanisms	Bacteria	Applications	Ref.
Rough surface +Nanozyme+NIR	Defect-rich MoS_2_	POD	ROS+PTT	*E. coli, S. aureus*	Wound healing	39
Rough surface +Nanozyme+light	Cu_2_WS_4_	POD +OXD	ROS	*E. coli, S. aureus*	*MRSA*-Infected wound healing	91
Rough surface +Nanozyme	Au@AgAu alloy	~	~	*E. coli, S. aureus*	Wound healing	104
Rough surface +Nanozyme	Carbon-iron oxide	POD	·OH	*E. coli, S. aureus*	Wound healing	30
Rough surface +Nanozyme	Hollow ceria nanoparticles	POD	ROS	*E. coli, S. aureus*	Wound healing	105

**Table 2:** Summary of cascade reactions in bacterial infection control

Strategies	Nanomaterials	Enzyme-like activities	Antibacterial mechanisms	Bacteria	Applications	Ref.
Cascade reaction	MOF	GOX peroxidase	ROS	*E. coli, S. aureus*	Wound healing	31
Cascade reaction	V_2_O_5_	haloperoxidases	HOBr+^1^O_2_	*E. coli, S. aureus*	Marine antifouling	73
Cascade reaction	GOX-Hb MRs	GOX + peroxidase	OH	*MRSA*	Antibacterial Agents	115
Cascade reaction	CaO_2_/H-G@alginate	Peroxidase	ROS	*E. coli, S. aureus*	Wound healing	116
Cascade reaction	GOX-on-Fe-iCOF	GOX + peroxidase	OH	*E. coli, S. aureus*	Wound healing	38
Cascade reaction	Au-Au/IrO_2_@Cu(PABA)	GOX + peroxidase	ROS	*E. coli, S. aureus*	Antibacterial Agents	117
Cascade reaction	L-Arg/GOX@CuBDC	GOX + peroxidase + Nitric oxide synthetase	ROS+RNS+NO	*E. coli, S. aureus*	Antibacterial Agents	118
Cascade reaction	ZIF-8@Au-GOX(ZAG)	GOX peroxidase	OH	*MRSA, S. aureus E. coli*	Wound healing	119
Cascade reaction	ZIF-8@GOX@BHb	GOX peroxidase	OH	*MRSA, ESBL E. coli*	Wound healing	120

**Table 3:** Summary of synergetic effects of metal ions and nanozymes for bacterial infection control

Strategies	Nanomaterials	Enzyme-like activities	Antibacterial mechanisms	Bacteria	Applications	Ref.
Ag^+^+Nanozyme+ light	Ag/AgBr/g-C_3_N_4_	OXD	ROS+Ag	*E. coli*	Water disinfection	132
Ag^+^+Nanozyme+ light	ZnO/Ag/RGO	OXD	ROS+Ag^+^	*E. coli, S. aureus*	Bacteria killing	142
Ag^+^+Nanozyme	Ag/Fe_3_O_4_	POD	^.^OH+Ag^+^	*E. coli, S. aureus*	Wound healing	143
Ag^+^+Nanozyme+ light	AgNPs/N-CD@ZnO	OXD	ROS+Ag^+^	*E. coli, S. aureus*	Wound healing	144
Ag^+^+Nanozyme	NH_2_-MIL-88B(Fe)-Ag	POD	^.^OH+Ag^+^	*E. coli, S. aureus*	Wound healing	28
Ag^+^+Nanozyme+ light	PCN-224-Ag-HA	OXD	ROS+Ag^+^	*E. coli, S. aureus*	Wound disinfection	33
Ag^+^+Nanozyme	MSN-Ag	OXD	ROS+Ag^+^	*E. coli, S. aureus*	Biofilm disinfection	35
Ag^+^+Nanozyme	GO/AgNPs/collagen	OXD	ROS+Ag^+^	*E. coli,S. aureus*	Bacteria killing	145
Zn^2+^+Nanozyme	ZIF-8@Au-GOX(ZAG)	POD+ OXD	Zn^2+^	*E. coli,S. aureus*	Wound disinfection	119

**Table 4:** Summary of synergetic effects of nanozymes and photothermal for bacterial infection control

Strategies	Nanomaterials	Enzyme-like activities	Antibacterial mechanisms	Bacteria	Applications	Ref.
Nanozyme+ NIR	PEG-MoS_2_	POD	ROS+PTT	*E. coli, S. aureus*	Wound healing	147
Nanozyme+ NIR	rGO-IONP	POD	·OH+ PTT	*MRSA*	Wound healing	148
Nanozyme+ NIR	Au@CeO_2_	POD	ROS	*E. coli, S. aureus*	Antibacterial Agents	135
Nanozyme+ NIR	Cu SASs/NPC	POD	·OH+ PTT	*E. coli, MRSA*	Wound healing	149
Nanozyme+ NIR	AuPtNDs	POD	ROS+PTT	*E. coli, S. aureus*	Antibacterial therapy	150
Nanozyme+ NIR	WS_2_QDs-Van@lipo	POD+OXD	ROS+ PTT	*Mu50, E. coli*	Anti-biofilm therapies	36
Nanozyme+ NIR	Au@Cu_2_-xS	POD	ROS+PTT	*E. coli, Faecalis*	Root canal therapy	154
Nanozyme+ NIR	RCF	POD	·OH+ PTT	*E. coli, S. aureus*	Wound healing	30
Nanozyme+ NIR	MoO_3_-x NDs	POD	·OH+ PTT	*E. coli, MRSA*	Wound healing	155
Nanozyme+ NIR	FePN SAzyme	POD	·OH+ ROS+PTT	*E. coli, S. aureus*	Wound healing	156
